# Electrochemical Nanosensors for Sensitization of Sweat Metabolites: From Concept Mapping to Personalized Health Monitoring

**DOI:** 10.3390/molecules28031259

**Published:** 2023-01-27

**Authors:** Riyanka Das, Somrita Nag, Priyabrata Banerjee

**Affiliations:** 1 Surface Engineering & Tribology Group, CSIR-Central Mechanical Engineering Research Institute, Mahatma Gandhi Avenue, Durgapur 713209, West Bengal, India; 2 Academy of Scientific and Innovative Research (AcSIR), Ghaziabad 201002, Uttar Pradesh, India

**Keywords:** electrochemical nanosensors, sweat metabolites, wearable sensors, real-time biosensing, personalized health monitoring

## Abstract

Sweat contains a broad range of important biomarkers, which may be beneficial for acquiring non-invasive biochemical information on human health status. Therefore, highly selective and sensitive electrochemical nanosensors for the non-invasive detection of sweat metabolites have turned into a flourishing contender in the frontier of disease diagnosis. A large surface area, excellent electrocatalytic behavior and conductive properties make nanomaterials promising sensor materials for target-specific detection. Carbon-based nanomaterials (e.g., CNT, carbon quantum dots, and graphene), noble metals (e.g., Au and Pt), and metal oxide nanomaterials (e.g., ZnO, MnO_2_, and NiO) are widely used for modifying the working electrodes of electrochemical sensors, which may then be further functionalized with requisite enzymes for targeted detection. In the present review, recent developments (2018–2022) of electrochemical nanosensors by both enzymatic as well as non-enzymatic sensors for the effectual detection of sweat metabolites (e.g., glucose, ascorbic acid, lactate, urea/uric acid, ethanol and drug metabolites) have been comprehensively reviewed. Along with this, electrochemical sensing principles, including potentiometry, amperometry, CV, DPV, SWV and EIS have been briefly presented in the present review for a conceptual understanding of the sensing mechanisms. The detection thresholds (in the range of mM–nM), sensitivities, linear dynamic ranges and sensing modalities have also been properly addressed for a systematic understanding of the judicious design of more effective sensors. One step ahead, in the present review, current trends of flexible wearable electrochemical sensors in the form of eyeglasses, tattoos, gloves, patches, headbands, wrist bands, etc., have also been briefly summarized, which are beneficial for on-body in situ measurement of the targeted sweat metabolites. On-body monitoring of sweat metabolites via wireless data transmission has also been addressed. Finally, the gaps in the ongoing research endeavors, unmet challenges, outlooks and future prospects have also been discussed for the development of advanced non-invasive self-health-care-monitoring devices in the near future.

## 1. Introduction

Personalized health care is vital to every individual and is allied with socioeconomic growth. The existing medical system is comparatively unreceptive and, typically, patients receive medical care after anguishing with comprehensible symptoms of a particular disease, despite the phenomenal advancement in the therapeutic arena. Therefore, the flawless trajectory of medical facilities is permitting health care staff to monitor health circumstances by initially evaluating health status and advising precautions against the suspected disease beforehand. In view of the above, currently, the modern health care system is rapidly growing worldwide, enabling at-home monitoring of health status [1].

Factually, there exist some characteristic limitations in the measurement of target-specific biomolecules from biofluids like blood, urine, tears, and saliva as monitoring of biomarker metabolites from blood specimens is invasive in nature. On the other hand, using urine is unfeasible within a wearable platform and tears from eyes cannot be easily used due to patient discomfort and eye irritation. Moreover, saliva may be intensely inflated by food consumption. As such, analysis of sweat can be the only potent option for the acquisition of biochemical information for dynamic health status monitoring in a non-invasive way [2]. Human sweat comprises numerous bio-analytes—ions, metabolites, hormones, proteins, etc.—which reflect the physical status of individuals. Detailed investigation of these explicit biomolecules as disease biomarkers has applied an extensive understanding of the health conditions based on real-time monitoring [3]. The concentration of these metabolites, owing to their noteworthy variations, can indicate the suspected diseases. The major metabolites that have been investigated for disease diagnosis include lactate, glucose, and alcohol. Concurrent investigation of such metabolites can deliver information for time-dependent health monitoring.

A number of health status-monitoring devices are available, such as stethoscopes and blood pressure monitors. However, these require expert personnel for operation and are also not feasible for the dynamic tracking of health status for a longer time period. On the other hand, electrochemical nanosensors are the most frequently employed sensors due to their standout advantages including high sensitivity, electrocatalytic behavior for target-specific detection, detection speed, cost-effectiveness and ease of synchronization with wearable devices [1]. Moreover, the key performance indicators for continued metabolite sensing by wearable devices, particularly for adapted domestic use, are flexibility, swift response, explicit sensitivity, inclusive linear range, a prompt signal-to-noise ratio, non-invasiveness, low-cost fabrication, on-body real-time data analysis, etc. [4]. Wearable biosensors can establish unified interaction with skin or clothes, enabling real-time dynamic monitoring of distinct health statuses, such as lactic acid and blood glucose levels [5]. Smart technological intervention in the domain of wearable sensors has empowered the miniaturization of bulky equipment with low-powered electronic circuitry and a flexible skin-friendly interface. Furthermore, the use of nanotechnology, through the incorporation of nanoparticles, carbon nanotubes, quantum dots, etc., in biosensors can competently increase the responsiveness of analytes by the sensor and improve transduction aptitude, resulting in amplified sensitivity of the sensor.

In recent decades, several review articles have been published on the electrochemical detection of sweat metabolites. Most of the review articles are focused only on the development of wearable sensor electrodes and the related technologies for wireless data transmission [6,7,8,9,10]. Some articles are oriented towards the detection of a single metabolite [11], while some are focused on the detection of sweat metabolites by a particular type of nanomaterial [12]. However, there is a lack of review articles for accumulatively streamlined comprehensive discussion regarding the basics of enzymatic as well as non-enzymatic sensor development for the electrochemical sensing of sweat metabolites followed by its integration with flexible substrates for fabricating wearable devices and an in-depth sensing mechanism under one umbrella.

**Figure 1:**
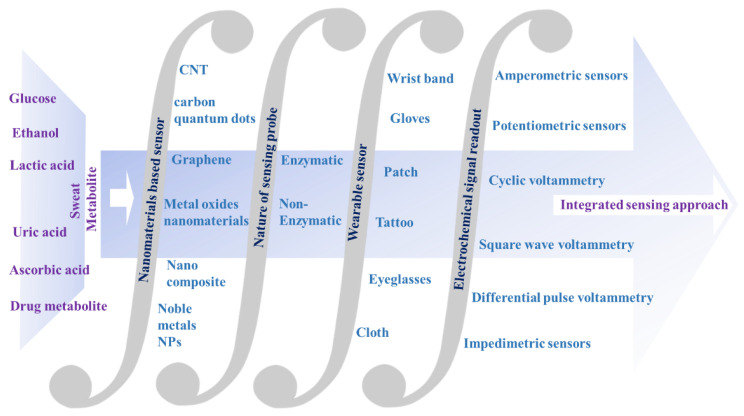
Schematic representation of the integrated approach for electrochemical nanobiosensors for sweat metabolite analysis.

In this context, the main aim of the present review is to offer a brief overview of current research endeavors in the electrochemical sensing of sweat metabolites, which act as important classes of disease biomarkers. Along with this, a detailed sensing mechanism with an in-depth discussion of sensing principles has also been presented. The integration of electrochemical nanobiosensors with suitable wearable electronics to develop user-friendly wearable devices has also been comprehensively detailed to acquire up-to-date information about the wearable sensing phenomenon in the realm of personalized health status monitoring (Figure 1). Apart from this, the unmet challenges of the recent research have also been briefly addressed for the judicious design of sensors with improved sensitivity.

In Section 2 of the present review, sweat production and varieties of sweat metabolites, acting as important disease biomarkers, have been summarized. In Section 3, the basics of the electrochemical sensing principles, including potentiometry, amperometry, cyclic voltammetry (CV), differential pulse voltammetry (DPV), square wave voltammetry (SWV), and electrochemical impedance spectroscopy (EIS), have been comprehensively reviewed for a fundamental understanding of the sensing phenomenon. These techniques exhibit the electrical signal change at the functionalized working electrode, which produces a readable signal to analyze the concentration of the targeted analytes. In Section 4, recent trends of (2018–2022) electrochemical sensing of sweat metabolites (e.g., lactate, urea/uric acid, glucose, ascorbic acid, ethanol, etc.) have been exclusively summarized. The in-depth sensing mechanisms, functionalization of the working electrodes with nanomaterials along with enzymes, sensitivities, detection thresholds and linear dynamic ranges of the sensors have been extensively scrutinized to acquire in-depth information about the sensing phenomenon. In Section 5, several non-implantable wearable devices in the form of several wearable platforms (e.g., eyeglasses, wrist bands, gloves, headbands, patches, clothes, etc.) have been acquisitively studied for monitoring metabolic biomarkers with an urgent emphasis on continuous health status tracking. Herein, the exploitation of a variety of flexible substrates, e.g., polyimide (PI), polydimethylsiloxane (PDMS), polyethylene terephthalate (PET), cotton, etc., has been summarized for fabricating flexible wearable devices to be worn on the human body, which can offer flexibility as well as conformability during various wearing circumstances. However, keeping in mind the structural complexity and tendency of interference from sweat metabolites during sensing, an additional notable approach has been introduced that describes the intermixing of the nanomaterials with the corresponding enzymes of target-specific metabolites for selective detection. This recent breakthrough in the fabrication of the aforementioned category of hybrid functional materials uplifted wearable sweat sensors as a preeminent contender in its category. The current breakthrough in the realm of fabrication of hybrid functional materials advances multifunctional smart wearable sweat sensors. Fundamentally, to investigate the practical implication of the sensor, several physicochemical parameters; for instance, chemical structures, electrochemical properties, interference from other metabolites along with performance-based aspects in varying environmental conditions; are to be considered. Therefore, in Section 6 and Section 7, the gaps and outlook of the present research endeavor along with future prospects have been briefly outlined for thoughtful designing of the sensor materials in the near future with desirable properties.

## 2. Analysis of Sweat Metabolites and an Initiative for Early Stage Disease Diagnosis

### 2.1. Sweat Production

**Figure 2:**
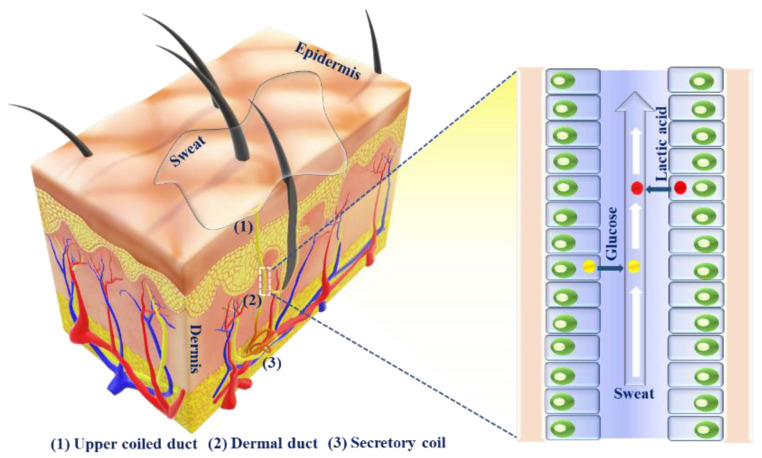
Schematic representation of eccrine sweat glands, sweat secretion pathway and biomarker partitioning, showing the metabolites, which are passed through the secreted sweat.

Sweat is a non-invasive disease diagnostic tool for monitoring important biomarkers related to detrimental diseases. Sweat can be engendered in the human body on an as-needed basis at the various locales of the body, e.g., chest, palm, neck, waist, forehead, sole, etc. [1]. Sweat glands are epidermic adjuncts that are normally distributed over the whole body, through which they are involved in perspiration. Sweat glands help in sweat secretion for maintaining body temperature, heat dissipation or as a response to emotions. Sweat glands may also act as excretory organs such as kidneys and lungs for drugs and their metabolites [13]. Sweat glands are primarily classified into two categories: one is eccrine and the other is apocrine. The former is more largely distributed across the entire body than the latter. Apocrine glands are located in hair follicles (e.g., scalp, groin, armpit, genital region, etc.) and mostly in private areas. Apocrine sweat is generated by adrenergic pathways and is mainly reliable on emotion, stress, etc., as it is sensitive to adrenaline [14]. On the other hand, eccrine sweat is secreted by the stimulation of nerves in cholinergic pathways. There are ~2–4 million eccrine sweat glands present in the human body, and these are mostly located in open skin surfaces, such as the palms of hands, soles of feet, forehead, etc. These contribute to the major part of sweat secretion across the whole body. The human eccrine sweat glands are comprised of secretory coils, dermal ducts and upper coiled ducts. The upper coiled duct is known as the acrosyringium, wherein there occurs a slight expansion of the secretory coil before its emergence on the skin surface [15]. After the production of sweat in eccrine glands, sweat is transported to the skin surface through dermal ducts and upper coiled ducts [16]. Important metabolites, such as glucose, urea, lactate, etc., from blood or generated locally through metabolic processes of the eccrine sweat glands, are partitioned through sweat, which provides valuable information about the body’s health status (Figure 2). The main content of sweat is water (∼99%). Sweat may be secreted in two ways: either in an active way or in a passive way. The continuous movement of muscles is responsible for active sweating, while passive sweating is caused by physiological or external factors, such as exercise, weather, etc.

### 2.2. Sweat Metabolites

Sweat can provide a wide array of biochemical information regarding the health status, as sweat contains a rich milieu of metabolites (e.g., glucose, urea, uric acid, lactic acid, ascorbic acid, creatinine, ethanol, etc.), which are the end product of a certain metabolic process [17]. Thus, these metabolites can act as important biomarkers for diverse diseases. In a disease state, sweat may contain different metabolites as biomarkers of the particular disease [13,18]. Thus, sweat comprises significant information, establishing it as the appropriate excretory fluid for diagnostic purposes. Glucose is a significant carbon-based component of sweat. Real-time screening of sweat glucose can provide information associated with diabetes mellitus [19]. Likewise, lactic acid/lactate is another important component of sweat. Lactic acid/lactate in sweat may be an important biomarker for stress ischemia, anabolism, cystic fibrosis, etc. The assimilation of lactate in muscles may result in muscle fatigue, discomfort, and soreness. Preeminent lactate concentration is a biomarker for muscle perfusion, and oxygenation and a valuable prognostic expedient for recovery in septic shock, due to inadequate oxygen delivery in tissue in case of hypoxia [20]. In addition, urea/uric acid is another important nitrogen-containing sweat metabolite, which is a key indicator of kidney disorders. In cases of patients suffering from kidney failure, the amount of sweat-secreted urea is greatly increased and can be found as a coating on white skin. Ascorbic acid (Vitamin C) induced procoagulant and prothrombotic activation of RBCs may increase the risk of thrombosis [21]. Thus, sweat-derived ascorbic acid may act as a biomarker of thrombosis. Orally administered ethanol is also excreted through skin perspiration and is a potential biomarker for an elevated blood alcohol level [22]. The main metabolites present in sweat, their concentration, and the related diseases have been summarized in Table 1. Analysis of sweat metabolites may be possible through the non-invasive collection of sweat from the dermal layers of skin containing large numbers of sweat glands. For analysis, sweat samples may be collected in two ways, either through a passive mechanism (e.g., physical exercise, such as running, cycling, etc., or by thermal stimuli, such as a foot bath) or by electrical stimulation (e.g., iontophoresis). Therefore, the collection of sweat followed by analysis of the abundant sweat metabolites may be a potential platform for non-invasive personalized disease diagnosis [23,24].

## 3. Detection of Sweat Metabolites: Initiative in the Context of Electrochemical Sensing Platform

Electrochemical techniques are surface techniques that are advantageous for the recognition of the bio-analytes present in sweat due to their standout advantages, e.g., profound selectivity, high sensitivity, rapid response, repeatability, low volume of sample requirement, cost-effectiveness, user-friendliness, portability, etc. In electrochemical techniques, recognition of the sweat metabolites followed by analysis of the targeted analytes are generally carried out via different electrochemical sensing modalities, such as amperometry, potentiometry, differential pulse voltammetry (DPV), square wave voltammetry (SWV), cyclic voltammetry (CV), electrochemical impedance spectroscopy (EIS), etc., which show the variation in electrical signals at the functionalized electrode, serving as a working electrode followed by transduction of the concentration of targeted analyte into readable signals [30,31].

**Figure 3:**
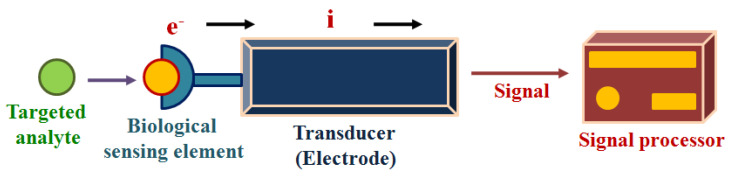
The working principle of the electrochemical biosensing.

Electrochemical sensors may be enzymatic or non-enzymatic. In the case of an enzymatic electrochemical sensor, the biological sensing element, as shown in Figure 3, is a particular analyte-responsive enzyme, which is responsible for the interaction of the targeted bio-analytes [32]. The chemical reaction between the immobilized bioreceptor on the working electrode with the targeted bio-analyte leads to the production or consumption of the electrons or particular ions, which ultimately affect the electrical behavior of the electrochemical sensor, such as the current or electrical potential [33]. Therefore, these techniques are used for the analysis of oxidation or reduction of the working system in the presence of electrical stimulus, and this redox reaction provides in-depth information about the concentration of the targeted analytes, reaction kinetics, reaction mechanisms and other important parameters. Thus, the electrochemical techniques can provide information about the selectivity, sensitivity and overall performance of the biosensors.

However, enzymatic electrochemical sensors are very sensitive to the surrounding environment, including factors such as temperature, pH, etc. Moreover, these kinds of sensors suffer from complications in storing, handling, external interference, etc. In addition, the background current may also be changed due to electrode surface fouling. To overcome these shortcomings, non-enzymatic sensors were developed, which generally utilize nanomaterials to improve the electrocatalytic behavior. The key requirements of the development of non-enzymatic electrochemical sensors are [34]:

1. The availability of the accessible active sites on the electrocatalytic sensor surface for adsorption and interaction of the active sites with the targeted analytes.
2. The capability of accessible active sites on the electrocatalytic sensor surface for engaging in multi-step electrocatalytic reaction.
3. Rapid charge transfer.

For the sensing of sweat metabolites, electrochemical sensors should possess several characteristics, such as rapid response, portability, detection accuracy, etc. In this perspective, chromo-fluorogenic optical sensors are explored for the recognition of sweat metabolite detection [35,36]. However, these suffer from severe restrictions, as optical techniques failed to provide a continuous response. Sweat turbidity has a major impact on the sensitivity of these kinds of sensors. Moreover, for quantitative analysis, a high-quality digital picture must be analyzed, which cumulatively restricts the real-time sweat analysis. On the contrary, due to the standout advantages (vide supra), electrochemical sensors can provide fast, continuous and real time data analysis to produce a sustainable solution for point-of-care monitoring of sweat metabolites.

As discussed earlier, depending upon the signal transduction, which may be the difference in current intensity, potential or impedance, electrochemical sensors may be categorized as amperometric, potentiometric and impedimetric, respectively [37]. Amperometric sensors, which rely on the measurement of current based on applied potential, are designated as voltammetric sensors.

### 3.1. Amperometric Sensors

Amperometry is an important electroanalytical technique for sweat metabolite detection. This can detect target-specific metabolites at threshold values ranging from 10^−6^–10^−7^ M. Amperometric sensors based on electron transfer among a substrate and electrode are capable for the development of sensitive and linear-responsive devices.

**Figure 4:**
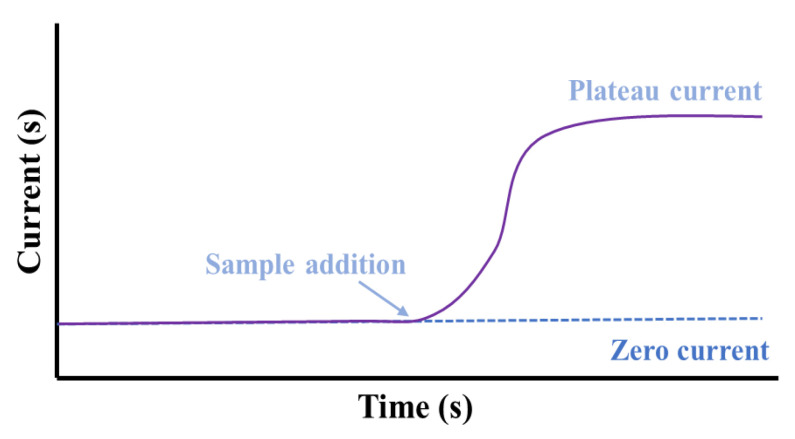
A response of an amperometric sensor indicating the increment in current at a particular potential with time after the addition of a sample.

Amperometry comprises the incessant oxidation or reduction process on the working electrode and quantification of the produced faradic current. The augmented potential is static during the measurement, and the generated current is noted over the progression of time (Figure 4). The electrochemical cell produces a weak current termed as the “zero current” without the addition of any sample. With the addition of a sample, the “zero current” situation is disrupted and the targeted species is transported through the electrolyte and is reduced at the electrode. Consequently, the current of this system increases up to a plateau indicative of equilibrium, and this “plateau reached current” is associated with the concentration of the targeted analyte to be detected [30]. Non-electroactive compounds can likewise be recognized indirectly by the amperometric method based on the ion-to-electron transduction mechanism [38,39,40]. This can be accomplished by means of electroactive conducting substance-modified electrodes, for which a specific amount of ionic species incorporation is needed to counterpoise the generated charge from the electron transfer reaction in the system. In another approach, the electrodes are modified with non-conductive and ion-exchange constituents, in which the electron transfer includes the exchange of the redox-active species by electrolytes and the generated current exhibits a direct proportionality with the concentration of the exchanged mediators. This process follows the diffusion-controlled path, and the recorded current can be related to the concentration of electro-inactive species in the solution.

Amperometry is advantageous over numerous controlled-potential techniques owing to the prompt sensitivity and selectivity for the detection of electroactive species, effectivity over a wide linear concentration range and by permitting the detection of analytes in adverse environmental circumstances. Furthermore, amperometric techniques have attracted much attention owing to the proficient detection capability of biomarkers from extracellular body fluids.

Electro-active species undergo electrochemical oxidation or reduction through the application of a potential that results in a steady-state anodic or cathodic current. The generated faradic current is proportional to the concentration of analytes. The reaction occurring at the working electrode (WE) by application of a specific potential might follow the below-mentioned mathematical equillibrium:

O_x_ + ne ⇌ R_ed_

The diffusion-controlled electron transfer is methodically represented as follows:

$$i=\frac{\mathrm{nFAD}(C_{\mathrm{bulk}}-C_{x=0})}{\delta }\;$$

where, i is the current, D is the diffusion coefficient, A is the surface area, n is the electron transfer number, F is the Faraday number, δ is the thickness of the diffusion layer and C_bulk_ and C_x = 0_ are the concentrations of analytes in the solution and in the electrode surface, respectively [41].

**Figure 5:**
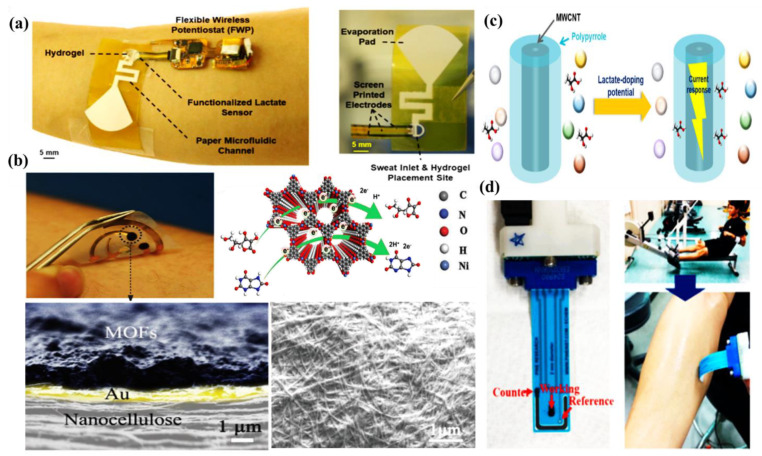
(**a**)OWLSS for continuous biochemical monitoring, adopted with permission from Ref. [42]; (**b**) Ni-MOF-based wearable sweat sensor, adopted with permission from Ref. [43]; (**c**) selective detection of lactic acid by MWCNT-polypyrrole nanowire; and (**d**) the fabricated flexible printed electrode, on which MWCNT-polypyrrole nanowires were impregnated.

Saha et al. have developed a wireless electrochemical continual sweat lactate measuring technique (Figure 5a) by utilizing a functionalized hydrogel for sweat extraction by osmosis, with a paper-based microfluidic device for the simplified transport of sweat. Herein, an electrochemical screen printed lactate sensor coupled with a potentiostat system has been attached to the microfluidic device, which provides prompt recognition responses at trace levels of sweat lactate with low power consumption and is monitored by the potentiostat. This osmotic wearable sensor for lactate recognition from sweat, entitled OWLSS, was further used for continuous measurement of sweat lactate transversely over workout activities with altering lactate concentrations [42]. Yang et al. have reported another sweat sensor for one-to-one health care through combining Ni-MOF (Ni_3_HHTP_2_)-based electrodes on the surface of a flexible nanocellulose substrate (Figure 5b). It comprehends the benefits of an exceedingly porous assembly, the characteristic electrical conduction property of the aforementioned Ni-MOF, skin compliance, and the higher mechanical strength of nanocellulose. The sensor device can conformably self-adhere to human skin for monitoring changes in sweat vitamin C levels during daily activities. This study validates the expansion of MOF-based flexible electrochemical devices in the realm of healthcare [43]. Choi et al. have developed a discerning non-enzymatic amperometric recognition technique for monitoring sweat lactic acid by using a multi-wall carbon nanotube (MWCNT)-polypyrrole core–shell nanowire (Figure 5c,d). Anions are doped in Polypyrrole (*p*-type conducting polymer), which leads to charge transfer, followed by discerning detection of lactate anions at definite potentials. Nanowires exhibited exceptional specificity to lactic acid over cohabiting interference of glucose, riboflavin, urea, etc., in sweat [44]. Cao et al. have reported a paper-based integrated microfluidic electrochemical device (3D-PMED) for the monitoring of sweat. Overall, 3D-PMED comprises five sheets: a sweat amasser, a vertical canal, a cross-wise canal, a layer of electrodes, and a perspiration disperser. The skin perspiration was captured with the assistance of a sweat accumulator and streamed through the vertical canal by means of capillary action. This 3D-PMED could efficiently adjust the sweat flow, which flawlessly resolved the challenging accumulation of sweat. The 3D-PMED was combined over a screen-printed sensor towards the investigation of a trace concentration of glucose in sweat [45].

### 3.2. Potentiometric Sensors

Potentiometry-based approaches are extremely significant in the arena of electrochemistry and are recurrently used for the recognition of diverse ions to numerous extents in environmental, agricultural, industrial, and also in therapeutic medication analysis. Recognizing an analyte is accomplished by electrochemical sensors through the generation of a particular signal equivalent to the concentration of the perceived analyte via an analytical device comprising a transducer. Conversely, these devices quantify the concentration of a particular analyte by monitoring the alteration in the potential among the working and reference electrodes within a particular detection range of 10^−8^–10^−11^ M [46,47,48].

Potentiometry measures the potential of the indicator electrode against a particular reference electrode. Herein, the indicator electrode remains in a straight interaction with the targeted analyte solution. However, a salt bridge separates the reference electrode from the analyte solution. The potential of the indicator electrode is proportional to the logarithm of the analyte activity in the solution.

Potentiometry deals with generated EMF in a galvanic cell wherein a spontaneous chemical reaction is occurring. To quantify the concentration of analytes in a sample, potentiometry considers the EMF response of a galvanostatic cell in an electrochemical cell potential under zero-current conditions. The below-mentioned Nernst Equation describes the response of such a cell:

EMF = K + RT/zF lna_1_

**Figure 6:**
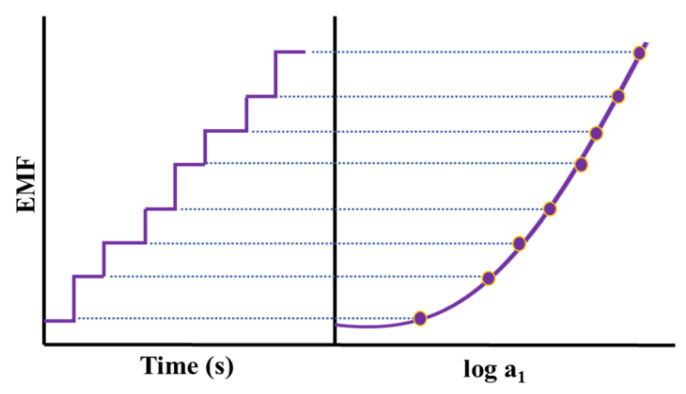
A dynamic response of a potentiometric sensor for increasing concentrations of an analyte with the calibration graph (logarithmic activity versus potential).

Here, EMF is the electromotive force (potential, observed at zero current), K is the constant potential contribution, which includes the liquid-junction potential at the reference electrode, a1 is ion activity consisting of charge z, and T, R, and F are the absolute temperature, gas constant, and Faraday constant, respectively (Figure 6).

Mou et al. reported a cuticular sensor that can wirelessly scrutinize metabolites by potentiometric pathway. They have fabricated an enzymatic ISE for potentiometric detection of sweat glucose. The ISE sensor is fabricated by amending a glucose oxidase coating (GOD) on an H^+^ ISE, which catalyzes glucose to produce H^+^. The as-produced H^+^ passes through the H^+^ discerning membrane to alter the potential of the electrode. They have completely inspected the LOD, and the stability of the cuticular sensor. Exploiting this potentiometric epidermal sensor, they have correlated the association between blood glucose and sweat glucose. The as-obtained results have indicated the concentration plot of sweat glucose, which is representative of the blood glucose concentration, which is beneficial for monitoring vital chronic diseases [49].

### 3.3. Voltammetric Sensors

Voltammetry-based methods are the most frequently implemented, specifically in the sensor domain. Cyclic voltammetry (CV) has arisen as the prime detection technique for the recognition of analytes. Voltammetry has been considered as one of the foremost electrochemical detection techniques for analyte detection. In this method, by varying the applied potential of the working electrode in both the anodic sweep and the cathodic sweep, the consistent current is measured. The applied voltage has numerous time profiles: linear voltage ramp (direct current polarography, linear sweep, and cyclic voltammetry); voltage step and voltage pulse application (chronoamperometry, chronocoulometry, reverse pulse polarography and voltammetry, and differential pulse polarography); and alternating voltage of minor amplitude superimposed on a linearly increasing voltage ramp (square-wave polarography, and voltammetry) [50].

#### 3.3.1. Cyclic Voltammetric (CV) Sensors

The concept of cyclic voltammetry is acquainted with the qualitative examination of the oxidation and reduction of a particular redox non-innocent system, which is a simple, reversible, diffusion-controlled electron transfer reaction. CV can also be implemented to quantify the rate of diffusion within a particular system. Cyclic voltammetry will be appropriate to study the electrochemical features of the system and the chemical reactions.

**Figure 7:**
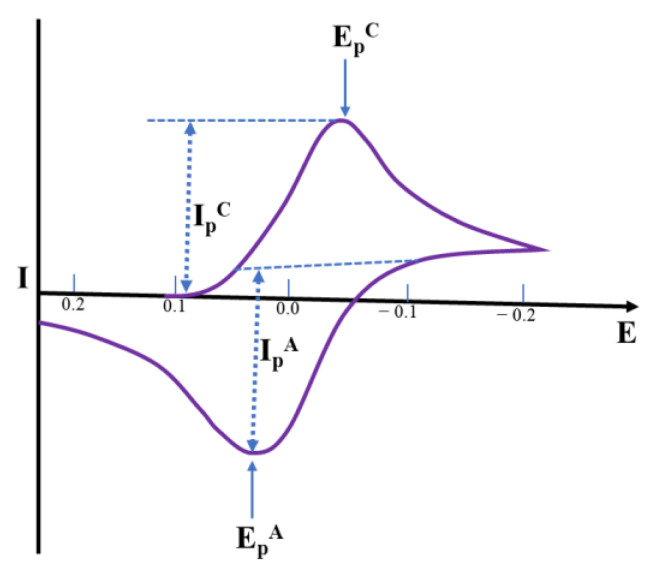
The shape of a cyclic voltammogram for a Nernstian electrochemical reaction, illustrating Current (I) Vs. Potential (E) plot, wherein the anodic peak current I_P_^A^ appears at the anodic peak potential E_P_^A^, and cathodic peak current I_P_^C^ arises at the cathodic peak potential E_P_^C^ for a reversible reaction.

Cyclic voltammetry is described through a sharp increase of potential at the working electrode within two potential limits. The potential limits and the sweep rate are straightforward adaptable parameters, and the characteristics of an electrolyte such as the concentration of a species may also be affected. In CV, the working electrode potential is swept across a potential range at a constant rate while measuring the resulting current. The potential is quantified within the reference electrode and the working electrode, and the current is quantified among the working electrode and the counter electrode. The three-electrode (working, reference, and auxiliary) technique is the utmost extensively used owing to the fact that the electrical potential of the reference electrode does not simply alter throughout the quantification. Depending on the surface negativity or positivity, a species may either acquire electrons from the surface or transfer electrons to the surface. This results in a quantifiable current in the electrode. The last characteristic of CV is intended to deliver information about the chemistry of redox non-innocent species. Such information is very useful in the detection of specific metabolites (Figure 7) [51,52].

#### 3.3.2. Differential Pulse Voltammetric (DPV) Sensors

**Figure 8:**
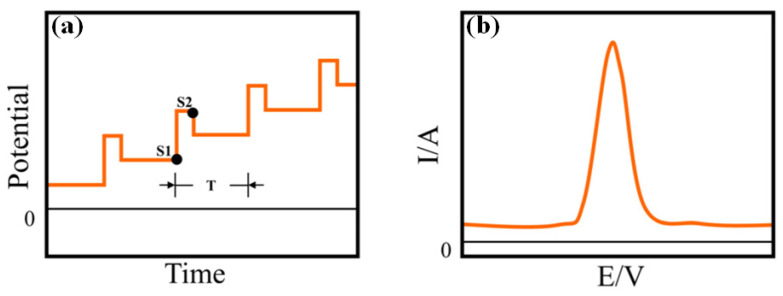

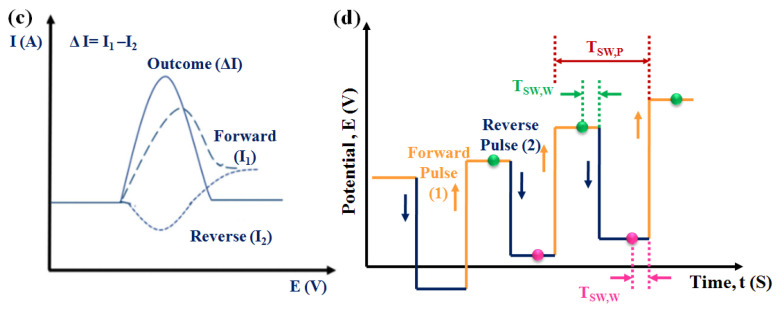
(**a**) The potential waveform and (**b**) the corresponding voltammogram of DPV, in the potential waveform, where T is the waveform period, and S1 and S2 are two current sampling points. (**c**) Schematic diagram of the application of potentials: sum of a square wave and a stair case and (**d**) schematic illustration of square wave voltammetry (SWV).

Differential pulse voltammetry (DPV) is another important voltammetric technique used for electrochemical detection of the targeted analytes. Herein, the supply of a base potential is required to the electrode where there is no Faradic reaction. Then, the base potential between the reference electrode and working electrode is gradually increased between equal pulse increments (Figure 8a,b). In this voltammetric method, the applied potential containing small pulses is superimposed on a staircase waveform. Measurement of the resulting current is immediately done before application of the pulse and at the end of the pulse. Accordingly, the difference of the resulting current for each pulse is also measured to obtain a relatively pure Faradic current. Herein, the difference of the current (I_S1_-I_S2_) is plotted as a function of potential to obtain a corresponding differential pulse voltammogram. Under normal conditions (with a pulse height of less than 100 mV), the peak height of the differential pulse voltammogram can be displayed as follows:

δtmax=nFAD12C*π12τ− τ′1−σ1+σ

where n is the electrons number, F is Faraday’s Constant (96,485 C/mol), A is the area of the electrode (in cm^2^), C* is the electroactive species concentration (in mol/cm^3^), D is the coefficient of diffusion (in cm^2^/s) and σ is exp (nFΔE/2RT), where T is the temperature (in K), ΔE is the pulse height and R is the Universal Gas Constant (8.314 J/mol⋅K). As pulse height is decreased, the quotient ((1 − σ)/(1 + σ)) is also decreased, tending to zero.

In this voltammetric technique, electrode reactions can be monitored more precisely, as herein only the Faradic current is extracted. Moreover, in this technique, a high sensitivity is obtained, as the effect of the charging current can be greatly minimized by measuring the current twice: before the pulse application and after the end of the pulse. The detection limit in this technique may reach up to 10^−8^ M. Due to its better sensitivity over CV because of its enhanced differentiation of the Faradic current, this technique is more desirable for the electrochemical recognition of the targeted biomolecules.

#### 3.3.3. Square Wave Voltammetric (SWV) Sensors

Square wave voltammetry (SWV) is one of the fastest and most sensitive voltammetric techniques for sweat metabolites detection. This may result in acquiring detection threshold values in the range of 10^−7^–10^−8^ M [53], close to those of the chronoamperometric technique, which is one of the most accurate electrochemical techniques.

In this voltammetric method, through the application of potential pulses, the corresponding current at the working electrode is sampled twice in each cycle, at the end of the forward square-wave pulse and at the end of the inverse square-wave pulse at a constant frequency. During the application of either the forward or reverse pulse, the sampling of the current is carried out at the same pulse period (T_SW,W_). So, if the total pulse period is designated by T_SW,P_, it includes both the forward as well as reverse pulses. Therefore, in the forward pulse, the current is sampled at 1/2 T_SW,P_-T_SW,W_, while the reverse pulse is sampled at T_SW,P_-T_SW,W_. Accordingly, the resulting current signal, which is actually the difference of inverse and direct currents, is acquired in the form of a ladder (Figure 8c,d). Therefore, the difference current is calculated as follows:

i_d_ = i_forward_ − i_reverse_

In this technique, each pulse is measured in such a way that the background current is highly minimized. SWV is a much less time-consuming process over other electrochemical techniques, as a SWV sweep can be recorded in less than ten seconds. Although, in most of the cases, for kinetic and mechanistic studies, CV is generally preferred over SWV, for a too-diluted solution, SWV is preferred due to its greater sensitivity. The core principle of SWV lies in the difference in charging (non-Faradic) and Faradic currents. Due to the exponential decay tendency, the decay of the charging current is more rapid than that of the Faradic current, which is inversely proportional to square root of time [54]. Therefore, at the end of each pulse, the capacitive current is rather insignificant with respect to the Faradic current [55]. This enhanced ratio of Faradic to capacitive current helps in lowering the detection threshold to enhance the sensitivity of the electrochemical sensors for the recognition of the targeted analytes.

### 3.4. Impedimetric Sensors

Impedimetric sensors measure the electrical impedance, which is generated at the interface of the sensor electrode by the supply of a small amount of the sinusoidal perturbation signal at the working electrode. Herein, application of a low amplitude of AC voltage is performed at the sensor electrode followed by measurement of the corresponding current response in steady state is carried out as a function of the frequency with the help of electrochemical impedance spectroscopy (EIS). The resulting current reflects the binding of the targeted analyte on the sensors interface, thereby quantifying the concentration of the analytes. EIS can give information about the different time constants, which are correlated to the different electrochemical processes, occurring at the sensor electrode interface. Moreover, EIS analyzes the variation of the electric charge transfer of any redox event, occurring at the working electrode. Generally, the current (I) is measured as a result of the application of disturbance to the potential (E). In the presence of the targeted bio-analyte on the sensor surface, the electron transfer resistance is enhanced. Therefore, the electrical resistance or impedance (Z) is the proportionality factor between E and I over a function of time, and Z is measured in Ohm, which is represented as follows:

$$Z=\frac{E\left(t\right)}{I\left(t\right)}\Omega$$

**Figure 9:**
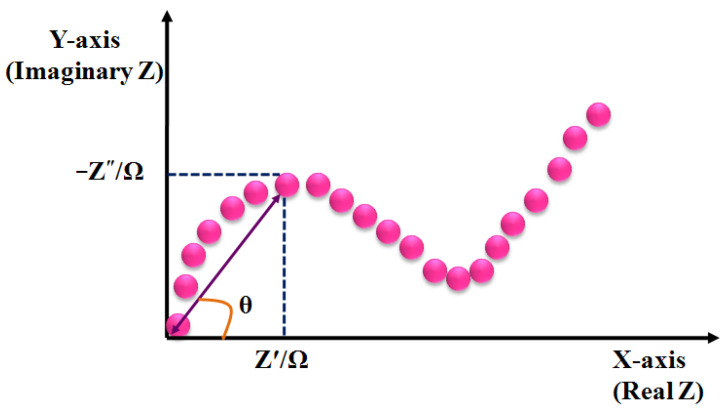
Schematic diagram of the Nyquist plot, illustrating the real (Z′) and imaginary components (Z″) of impedance.

The impedance can be analyzed using Nyquist plot, where the semi-circle region of higher frequencies indicates the controlled behavior of the charge transfer. The increasing size of the semicircle indicates the increase in the charge transfer resistance [56]. The straight line at lower frequencies after the semicircle represents the diffusion-controlled behavior (Figure 9). Therefore, EIS is one of the most effective techniques for the analysis of interface phenomena, e.g., electric double layer, diffusion, etc. It does not require any chemical label, as the variation of impedance can be acquired by binding the targeted analyte on the sensor surface. However, the major drawback of this technique is the intricate post-processing process, which lessens the sensitivity of the sensor surface.

## 4. Nanomaterials-based Electrochemical Sensing of Sweat Metabolites

The advancement of nanotechnology along with the surface functionalization processes allows miniaturized devices for providing highly selective and sensitive measurements of the targeted bio-analytes present in sweat. Recently, non-enzymatic electrochemical sensors have gained major research interest. Currently, chemically modified electrodes are widely used in place of bare electrodes to enhance the selectivity as well as sensitivity of the electrochemical sensors. To acquire more stability, repeatability, simplicity, etc., these types of sensors utilize diverse nanomaterials, as nanomaterials have the capability to highly enhance the electrocatalytic properties of electrochemical sensors because of their large surface area, available active sites, unique nanoscale morphologies as well as enhanced electron transportation by reducing the interfacial resistance. Carbon-based nanomaterials [57] (e.g., CNT, carbon quantum dots, graphene, etc.) and noble metals (e.g., Au, Pt, etc.) are used as efficient conductive support materials to modify the working electrodes, thus improving the electrocatalytic behavior of the electrochemical sensor. Metal oxide nanomaterials (e.g., ZnO, MnO_2_, NiO, etc.) are used in electrochemical sensing due to their ultralow size, good electrical and chemical behavior, high catalytic behavior as well as cost-effectiveness. Sometimes, composite materials are also used for further improvement of sensitivity of the electrochemical sensors. Owing to the improved electrical and chemical properties along with mechanical robustness, nanomaterials-based electrochemical sensors are much more preferred over the bulk materials. The recent advancement of nanomaterials-based electrochemical sensing of sweat metabolites (glucose, urea/uric acid, lactic acid/lactate, ascorbic acid, ethanol, drug metabolites) has been discussed in this section (Table 2).

### 4.1. Glucose @ a Major Biomarker for Diabetes Mellitus

Diabetes mellitus is a life-threatening enduring disease. The elevated levels of blood glucose (BG) can generate an extensive assortment of health issues. Therefore, it is highly crucial to monitor BG levels as preventive care for issues in diabetes monitoring. The foremost biological fluid selected for glucose level measurement is blood, in which finger pricking is the most commercial and universal technique. However, finger pricking causes unavoidable pain, skin annoyance and microbial contagions. Therefore, glucose monitoring in a non-invasive way has been considered recently. A plethora of non-invasive glucose monitoring methods has been developed. However, the outcomes of these previously invented methods have been proved to be comparatively less effective owing to particular physico-chemical interference factors. Besides researching a new non-invasive method [58,59], detection from different body fluids, including sweat, tear, saliva, follicles, exudates, etc., has also been investigated. Glucose can be found in the range of 0.1–50 mg/dL in human sweat. Hence, non-invasive recognition of free glucose from sweat can be considered as an alternative way of measuring blood glucose levels invasively [60,61].

**Figure 10:**
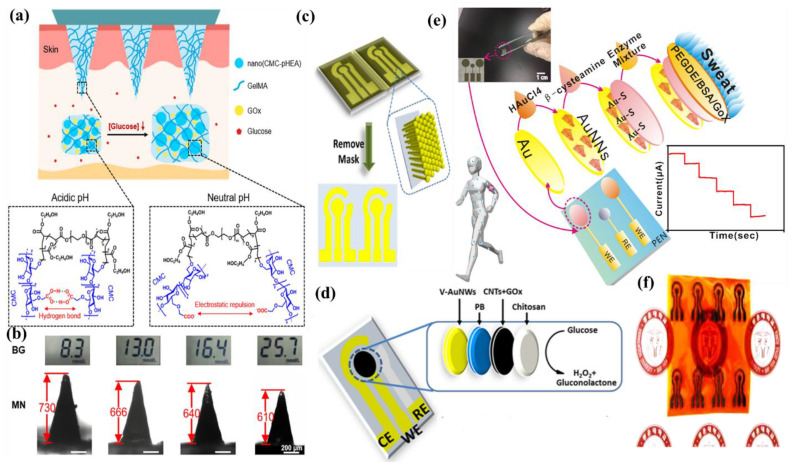
(**a**) Schematic illustration and responsive mechanism of a microneedle system for glucose monitoring; (**b**) CCD images of the microneedles after penetration into the skins of mice with BG levels; (**c**,**d**) fabricated v-Au NW-based stretchable electrodes on flexible substrates, adopted with permission from Ref. [61]; (**e**) schematic diagram of the flexible and wearable electrochemical sensor for glucose and lactate detection, adopted with permission from Ref. [57]; and (**f**) fabricated LIG electrode on polyimide (PI) film of the proposed enzymatic glucose sensor, adopted with permission from Ref. [63].

Wang et al. have invented a dual-layered incrusted microneedle arrangement for less-invasive measurement of trace levels of glucose (Figure 10a) [60]. The microneedles exhibited adequate mechanical strength to infiltrate through the skin of the mice model and allowed an in-situ glucose-receptive reaction. The microneedles exhibited prompt receptiveness to pH and glucose on skin-imitating gels and in diabetic mice models (Figure 10b). Mechanistically, the sweat glucose levels can be quantified by the ratio of alteration in the heights and swelling of microneedles, and the readouts were comparable with the measurements by the glucometer. The detection of glucose through the microneedle array system follows the glucose oxidase (GOx) catalyzed reaction of glucose and oxygen to form gluconic acid, therefore producing an acidic environment. By exploiting this reaction, the GOx-loaded microneedle array system exhibited sensitive selectivity towards diverse glucose concentrations. This microneedle array delivers a prompt, minimally invasive, and consistent glucose quantification platform for the diagnosis of diabetes.

Zhai et al. have synthesized elastomer-bonded Enokitake-mimic gold nanowires (v-Au NWs), modified by glucose oxidase (GOx) and Prussian blue (PB) nanoparticles, which can be strained equal to 800% devoid of a trailing conduction (Figure 10c,d) [61]. They modified the working electrode with vertically aligned Enokitake-like gold nanowires (v-Au NWs). Those chemically modified electrodes were utilized in a 3-electrode system, wherein unmodified v-Au NW and Ag/AgCl were used as counter and reference electrodes, respectively. Herein, PB was used as a working electrode and acted as a substitute for peroxidase, thus improving the feasibility of low-potential glucose detection. In CV, the distinctive oxidation-reduction points of PB at 0.15 and 0.1 V were obtained. However, after glucose addition in a significant amount, the oxidation peak diminished and the reduction peak was augmented, signifying the prompt sensitivity of the sensor toward the recognition of glucose. The LOD of the glucose-sensing phenomenon was found to be 10 µM. The sensing of glucose was investigated with varying lengths of the working electrode with artificial sweat samples, with the achievement of sensitivity as 4.55 µA•mM^−1^•cm^−2^. Straining could be detected throughout the electrochemical reaction concurrently by 2.30 gauge factor and by 22.64 as obtained from chronoamperometric results. For 3 stages of applied strain levels of 10%, 20%, and 30%, with successive addition of glucose, uniform staircase-like current vs. time chronoamperometry curves were observed, which signified the accuracy of glucose detection in the range of 0–800 µM under a deformative condition.

Yu et al. have fabricated a flexible sensor for prompt monitoring of micromolar level glucose by using gold nanopine needles with a signal intensification approach (Figure 10e) [62]. Gold nanopine needles (AuNNs) were electrochemically deposited on a working electrode. Furthermore, on the electrode surface, an infusion of GOx was executed via a cross-linking process by a noteworthy constituent of redox hydrogel, poly (ethylene glycol) diglycidylether (PEGDE), proficient for the retention of the GOx activity. Mechanistically, glucose is oxidized by GOx to generate H_2_O_2_ by applying potential on the electrode. In this process, disintegrated H_2_O_2_ produces H^+^ ions and electrons, which result in significant alteration in current, thereby exhibiting the concentration of sweat glucose. Being an enzymatic sensor, the glucose detection ability increases with the enhancement of the enzyme’s catalytic capacity. The as-developed sensor exhibits the LOD of glucose as 7 µM. Furthermore, a real field application was accomplished via the detection of glucose from different sweat samples.

Yoon et al. have developed an acetic acid surface-modified laser-induced graphene (LIG) electrode via a simplistic dipping technique (Figure 10f) [63]. The acetic acid treatment intensely amplified the carbon–carbon bonds ratio and reduced the carbohydrate functional groups, which effectually augmented the conductivity and facilitated the stable dispersity of Pt nanoparticles (PtNPs) on LIG by preventing aggregation owing to the electric field on the nanoparticles. Lastly, a chitosan–glucose oxidase (GOx) composite was efficaciously impregnated onto the LIG/PtNPs electrode to construct a biosensor for monitoring sweat glucose. Mechanistically, GOx helped in the oxidation of glucose to produce glucono-δ lactone as by-product. Concurrently, there occurred a self-reduction of GOx, which again transforms oxygen to H_2_O_2_. PtNPs oxidize H_2_O_2_ into oxygen and produces two electrons, which resulted in an amperometric response under a positive potential.

**Figure 11:**
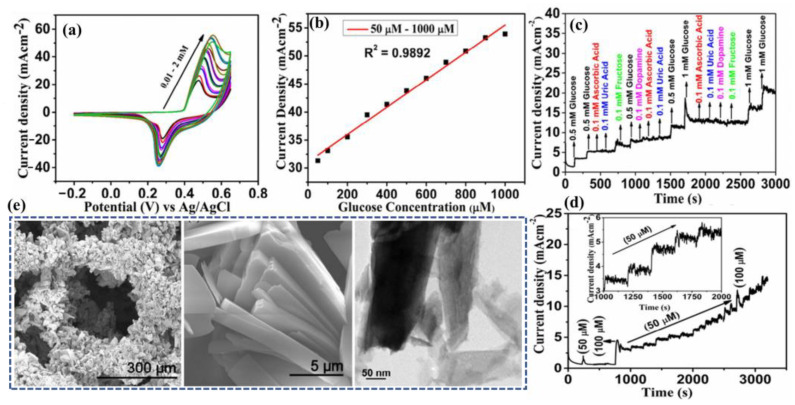
(**a**) CV curves at different glucose concentrations, (**b**) derivative plot indicating the increase of current with glucose concentration, (**c**) interference study: amperometric response of the electrode upon successive addition of glucose, ascorbic acid, uric acid, dopamine, (**d**) amperometric response upon addition of glucose, and (**e**) SEM and TEM images of the fabricated nano-flakes layer on nickel foam. Adopted with permission from Ref. [64].

Padmanathan et al. have fabricated a nano-flakes layer of Ni_3_(PO_4_)_2_•8H_2_O on nickel foam (NF) through a hydrothermal method and examined the newly developed electrode towards a sweat-based glucose sensor [64]. The Ni^3+^ species catalyze the deprotonation and isomerization of glucose in the electrode surface. This results in the diffusion and subsidence of glucose intermediates into the bulk solution and the subsequent rejuvenation of the active sites. Ni^2+^ is attached to phosphate with oxygen atoms, wherein the chance of bonds reorganization was possible by electro-oxidation of glucose at a diverse kinetic rate, significantly improving both the cathodic and anodic peak currents (Figure 11). The non-enzymatic glucose sensor, Ni_3_(PO_4_)_2_•8H_2_O/NF, exhibited an extraordinary sensitivity with a very low detection threshold of 97 nM.

### 4.2. Urea/Uric Acid @ a Major Biomarker for Renal Dysfunction

Uric acid (UA) is found mostly as urate at biological pH. The usual UA level in a healthy person lies in the range of 300–500 µM in plasma, 100–250 µM in saliva, and 18–35 µM in sweat. The elevated level of UA causes inflammation for gout, renal dysfunction or neuroprotection tendency. However, lower levels of UA are disadvantageous to neurons, as UA exhibits antioxidant properties in physiological conditions and supports the reinforcement of the body’s free radicals’ attack and defense. As a consequence, non-invasive electrochemical nano-sensor-based detection of UA with high sensitivity and a low operating range is of immense importance, being an easy-to-monitor aid in comparison to that of any previously developed invasive technique [65,66,67].

Azeredo et al. have developed a screen printed electrode (SPE) utilizing alpha nickel hydroxide nanoparticles (α-Ni(OH)_2_ NPs) with improved activity of electrocatalysis for the oxidation of uric acid via incorporation of Zn^2+^ by a sol-gel technique [67]. The appropriateness of constructing inimitable sensors was established by means of batch injection analysis (BIA) integrated with modified FTO (fluorine-doped tin oxide) electrodes with LOD of 2.28 × 10^−8^ M. The α-Ni_0.75_Zn_0.25_(OH)_2_ material was effortlessly combined in the ink, which provided promising conductivity and mechanical stability to the films, restricting their solubility in neutral and mildly acidic conditions. Voltammetric and chronoamperometric investigations were performed based on SPEs equipped with graphite ink incorporating α-Ni_0.75_Zn_0.25_(OH)_2_ nanoparticles. A Ni(OH)_2_-based electrocatalyst in mildly acidic conditions and effective transformation to screen-printed sensors help in easy upscaling. CV indicates the shifting of the peak potential to more positive values as pH diminished, corroborating with a plausible contribution of protons in UA oxidation reaction.

### 4.3. Lactic Acid/Lactate @ a Major Biomarker for Stress Ischemia

Lactic acid (LA) is one of the leading biomolecular entities extensively distributed throughout the human body. Elevated levels of LA are associated with health-hazardous diseases. The quantification of LA within blood levels is of utmost necessity for the possible diagnosis of stress ischemia, head rupture, and cardiovascular diseases such as hypoxemia and anemia. LA is the potential biomarker for energy metabolism and tumorous cells. For example, elevated levels of LA can be observed in brain tumor cells. Consequently, for health assessment, regular screening of the LA level is crucial. However, the detection of blood lactic acid is invasive in nature, causing inevitable pain and skin annoyance. On the other side, sweat contains a significant level of LA, which is elevated as an indication of a bio-marker for a particular disease. Therefore, sweat can be a promising alternative to blood for monitoring LA levels [68,69,70,71].

**Figure 12:**
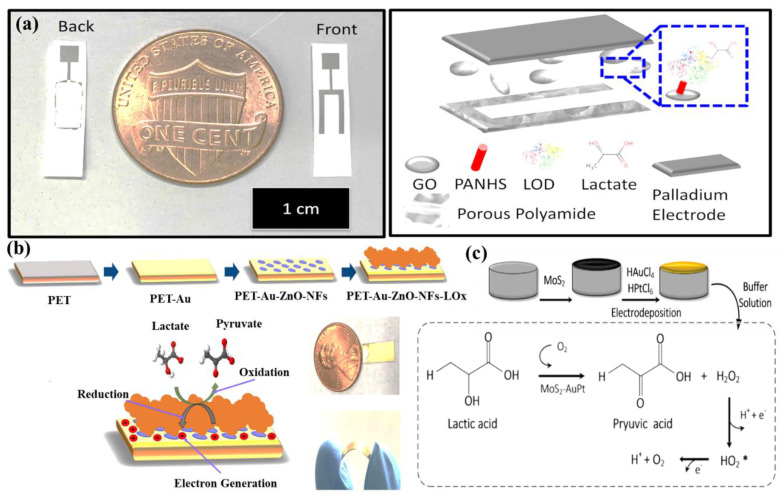
(**a**) Drawing of the graphene oxide through a membrane sensing platform for electrochemical affinity-based detection of lactate, adopted with permission from Ref. [72]; (**b**) PET/Au/ZnO-NFs-based electrode and flexibility of PET/Au/ZnO-NFs electrode; and (**c**) detection of LA using MoS_2_-AuPt@SPE, adopted with permission from Ref. [70].

Lin et al. have developed an impedimetric sensor for sweat lactate at low volume (1–5 µL), which is comprised of graphene oxide (GO) nanosheets unified on a nano-porous flexible electrode (Figure 12a) [72]. Lactate oxidase (LOx) was infused in the periphery of GO for improving the selectivity of the biosensors within the range of 4 to 80 mM of sweat lactate. Herein, lactate detection was accomplished based on the production of ions in the LOx-catalyzed enzymatic reaction, which produces an alteration in resistive behaviors and was monitored by EIS. The sensor exhibited a working range of 1–100 mM in sweat with a LOD of 1 mM. The sensor was receptive to concentrations of sweat lactate up to 138.6 mM.

Alam et al. have developed a highly selective, sensitive, and flexible enzymatic sensor based on zinc oxide nanoflakes (ZnO NFs) for non-invasive lactic acid (LA) sensing from sweat (Figure 12b) [71]. Synthesis of ZnO NFs on gold-coated flexible polyethylene terephthalate (PET) substrates was carried out by sonochemical process. Lactate oxidase enzyme (LOx) was infused on the ZnO-NFs-based electrode, which was highly selective for lactate sensing. Herein, LOx, acting as an electrocatalyst, permitted the electrons to flow within the substrate and the electrode surface comprised of no intermediate layer. The output signal of the sensor changed due to the redox reaction by oxidation of LA in the presence of LOx and the consequent formation of pyruvic acid. The sensor was examined in the range of 10 pM–20 mM, which covered the physiological range. The LOD value was obtained as 1.26 nM. The response of the highly reproducible, linker-free sensor was 55 times better in comparison to the conventionally used linker-infused gold electrodes due to having a significantly high isoelectric point of ZnO-NFs.

Xiao et al. have developed a non-enzymatic sensor for the quantitative detection of LA based on MoS_2_-AuPt nanocomposite-modified SPE (Figure 12c) [70]. MoS_2_-AuPt nanocomposites facilitated the electron transfer and AuPtNPs exhibited exceptional electrocatalytic oxidation ability to LA, proving the suitability for LA detection from sweat samples. The SWV curves indicated gradual peak current shifting with the increase of LA concentration, indicating oxidation of LA by the electrode. The non-enzymatic LA sensor exhibited a low LOD of 0.00033 mM, a fast response time of 15 s, high sensitivity, and a wide linear range. Owing to the modest operational strategy, portability and low cost, the developed sensing platform was predicted to combine with a microneedles-based electrochemical biosensor in the near future.

Liu and Li et al. have described an electrochemical sensor based on TiO_2_ nanotube arrays and Ti mesh composite for sweat glucose, urea and lactic acid sensing [73]. Herein, the nanotube facilitated deeper penetration into the pores for sweat absorption, while the Ti mesh helped in gas permeability for avoiding skin damage. From the cyclic voltammogram data, it has been observed that with increasing concentrations of urea and lactic acid, the response current was gradually increased, while for glucose, the response current was gradually decreased. The sensing mechanism was elaborated based on density of states (DOS) calculations, which exhibited that the narrowing of the band gap is responsible for the generation of the current response. However, the glucose sensing mechanism has not been fully explained herein, which requires further research.

Yu et al. have fabricated a flexible sensor for the micromolar-level detection of sweat lactate by means of gold nanopine needles (AuNNs) in a signal intensification technique [62]. AuNNs were dropped on the working electrode, and the enzyme was infused on the electrode surface via cross-linking of poly (ethylene glycol) diglycidylether (PEGDE), which is a significant constituent of redox hydrogels for retaining the enzyme activity. The catalytic behavior of AuNNs was investigated under diverse conditions. Mechanistically, lactate is oxidized by LOx to generate H_2_O_2_ by applying the potential on the electrode. The decomposition of H_2_O_2_ led to the formation of H^+^ and electrons, which led to variation in the current, and therefore indicated the concentration of glucose in sweat. The present sensor was found to accomplish a LOD of 54 µM for lactate. The selective and reproducible sensor analyzed sweat samples, which indicated its plausible application in determining lactate with a wearable skin sensor.

### 4.4. Ascorbic Acid (AA) @ a Major Biomarker for Kidney Disease

Ascorbic acid (AA) is an important water-soluble vitamin, playing an essential dietary component in the human body. Like other water-soluble vitamins, it is not accumulated inside the body for an extended time period and therefore is excreted through body fluids, like sweat on a timely basis [24,74]. It plays an important role to restore the immune system of the body, wound healing and also to improve the antioxidant capacity of the body. By scavenging the free radicals inside the body, AA can minimize the growth rate of cancer cells inside the body [75]. AA is also clinically utilized for treatment of hematological diseases, cancers, tumors, anti-aging, stones, etc. [76]. However, prolonged consumption of AA may lead to kidney disease, thrombosis, cancer, etc. This necessitates the regular screening of AA from body fluids such as sweat as a part of non-invasive personalized self-health monitoring for the prevention of vitamin imbalance in the body and related disorders.

**Figure 13:**
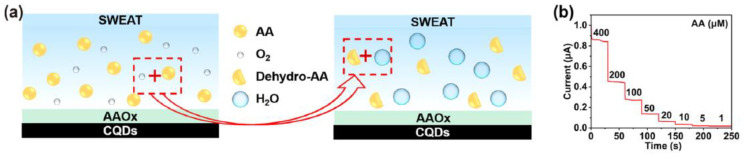
(**a**) Schematic illustration of the mechanistic pathway of sensing, (**b**) amperometric response of the sensor in presence of 1–400 µM AA in artificial sweat, adopted with permission from Ref. [77].

There are a good number of literature reports where AA can be detected by enzymatic sensors. Recently, Zhang et al. have reported a portable sweat sensor comprised of six electrodes based on carbon quantum dots for selective and sensitive recognition of AA [77], where the recognition phenomenon proceeds through an enzymatic catalytic reaction. CQD has been chosen as the electrode substrate material of the electrochemical sensors for immobilization of enzymes because of their large surface area [78] and abundant active sites for effectual reduction of the interfacial impedance to increase the sensitivity of the sensor [79]. For AA detection, herein ascorbic acid oxidase (AAOx) has been immobilized onto the electrode material, which was confirmed by the increase of the charge transfer resistance (R_ct_) from 36.1 to 184.4 kΩ, which was further enhanced to 276.0 kΩ after cross-linking by glutaraldehyde. The enzyme catalytic reaction proceeded via oxidation of AA by consumption of oxygen, which consequently led to the reduction of oxygen, of which the potential was measured to be 0.4 V by CV (Figure 13a). It has been observed from the amperometric response data (Figure 13b) that increasing the concentration of AA is linearly correlated with the response current, which was produced from enzymatic reaction.

**Figure 14:**
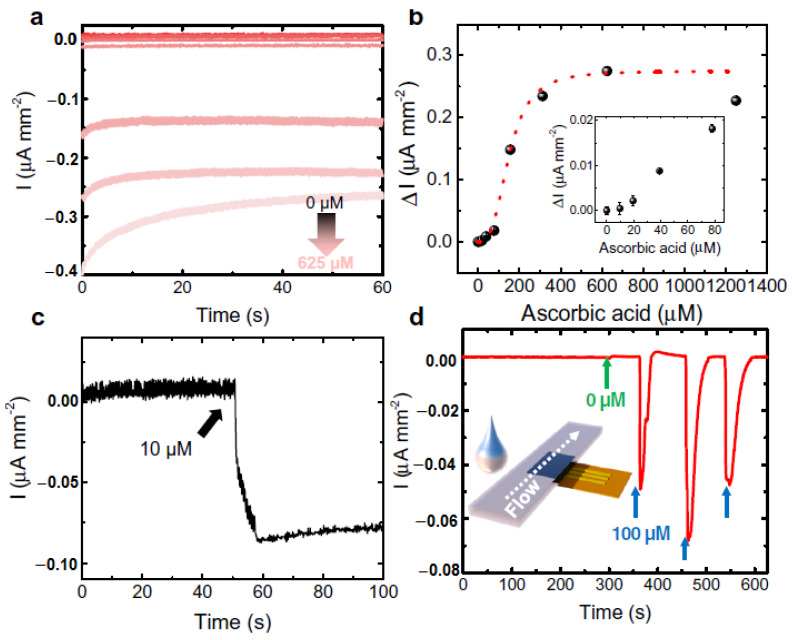
Determination of ascorbic acid by gold microelectrode-based flexible membrane biosensor from artificial sweat solution. (**a**) Chronoamperometry and (**b**) calibration at 5 mV vs. Au pseudo ref. electrode. Inset enlarged view at the lower concentrations, (**c**) real-time chronoamperometric measurement of 10 µM ascorbic acid (AA) by direct drop casting, and (**d**) real-time measurement of sweat in the presence and absence of AA by a filter paper adopting lateral flow approach.

However, these kinds of enzymatic sweat sensors suffer from several shortcomings. One of those is that these cannot be easily integrated with electrochemical sensors due to certain restrictions, such as pH, temperature, etc. [80]. To overcome the shortcomings, a gold microelectrode-based flexible membrane biosensor has been reported by Ibarlucea et al. for electrochemical AA sensing from sweat samples [81]. The gold microelectrode was formulated on a polyimide substrate and then an alginate membrane was electrodeposited onto it with encapsulated CuO nanoparticles (NPs). The developed biosensor can detect AA based on its oxidizing capability of CuO NPs. Initially, cyclic voltammetric and amperometric determination of AA has been carried out in a buffer solution at a fixed pH. Ascorbate produced from ascorbic acid led to the shifting of the redox peak due to migration of CuO NPs through variable oxidation states. The shifting of the redox peak increased linearly with the concentration of AA. The biosensor can detect AA in the micromolar range with the sensitivity of 0.103 V log (µM)^−1^ of the peak shift. It has also been exploited towards recognition of AA in artificial sweat samples with high selectivity and sensitivity, performing the chronoamperometric measurements at −5 mV vs. Au pseudo-reference electrode (Figure 14a–c). For the application of the designed biosensor as a potential wearable device, a lateral flow approach has been adopted by combining the sensor onto a filter paper, which also produced an obvious redox signal in the presence of AA (Figure 14d). Though it is susceptible to ascorbic acid detection in artificial serum samples, more research is needed to improve the performance of the sensor for on-time detection of AA from real sweat samples with the optimization of the design of the portable miniaturized device.

**Figure 15:**
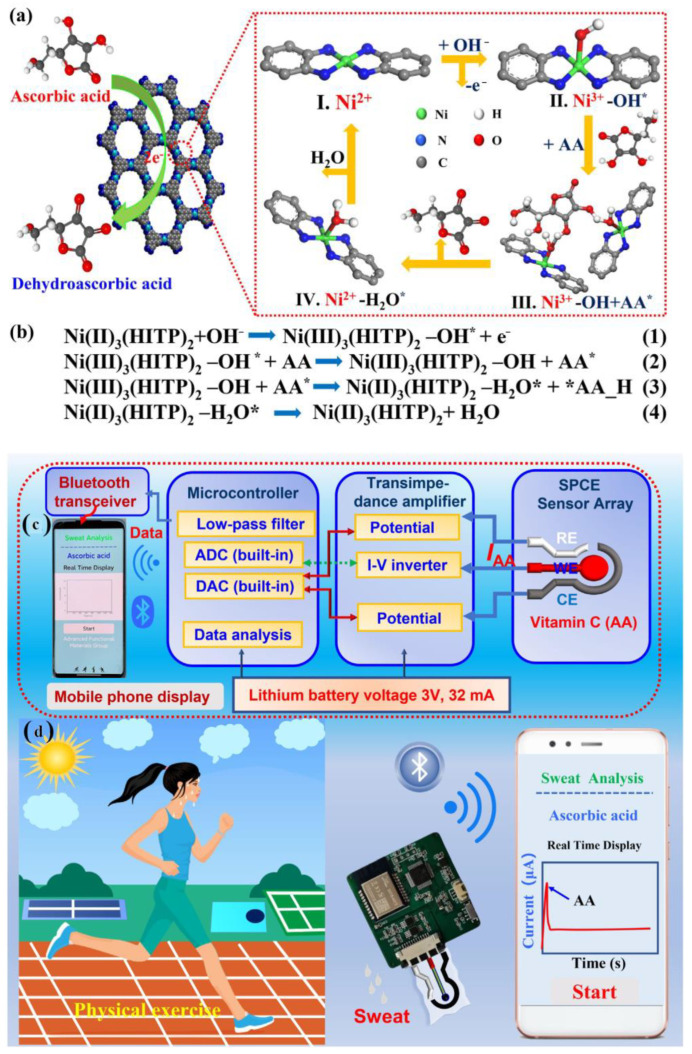
(**a**) Plausible reaction pathway of electrocatalytic oxidation of AA by Ni_3_(HITP)_2_, (**b**) the plausible equations of the corresponding catalytic reactions on Ni-MOF/SPCE surface (where * denotes the formation of activated species), (**c**)schematic diagram of portable AA sensing system and (**d**) the corresponding health monitoring system integrated with a smartphone app, adopted with permission from Ref. [82].

Yu and Hou et al. have reported a non-enzymatic electrochemical sensor comprised of conductive nanorods of Ni-MOF (Ni_3_(2,3,6,7,10,11-hexaiminotriphenylene)_2_; Ni_3_(HITP)_2_) made screen-printed carbon electrode (SPCE) for on-site detection of AA [82]. The pores of the Ni-MOF with a diameter of ~1.5 nm may be responsible for the adsorption of AA onto the MOF, which is responsible for the electrocatalytic oxidation of AA at the active catalytic sites, leading to the generation of a higher oxidation peak. During the process of electrocatalysis, SPCE remained almost inert. However, no subtle change was perceived when a control electrocatalytic oxidation study was carried out with Ni/SPCE, which attests to the key role of Ni(II) in the conductive Ni-MOF towards electrocatalytic recognition of AA. In alkaline conditions, Ni(II) was interconverted to Ni(III), which catalyzed the oxidation of AA to dehydroascorbic acid (DHAA, C_6_H_6_O_6_) (Figure 15a,b). With the gradual increase of AA concentration, the oxidation peak potential shifted to higher values in the linear range of 2–200 µM. Accordingly, the limit of the detection value was discovered to be 1 µM. To further support the electrocatalytic reaction, density functional theory (DFT) studies were also performed to study the interfacial charge transfer during the electrocatalytic reactions. Based on the experimental outcomes, herein a portable device wirelessly integrated with a smartphone has been fabricated for real-time AA sensing in human sweat (Figure 15c,d).

**Figure 16:**
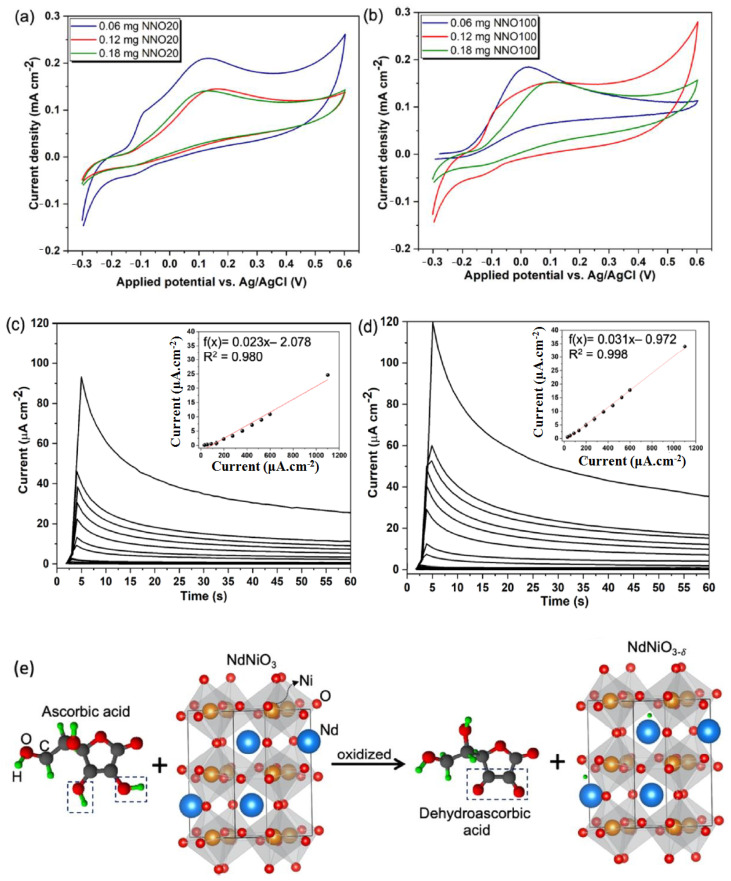
(**a**) Cyclic voltammetry of GO/NNO20 and (**b**) GO/NNO100 nanotubes in the presence of 1 mM of AA; chronoamperometric data of (**c**) GO/NNO20 and (**d**) GO/NNO100 in the presence of increasing AA concentrations (30–1100 µM) in PBS electrolyte. Applied potential: +0.1 V; (**e**) plausible sensing mechanism of AA by NNO via oxidation of AA to DHAA and reduction of Ni^3+^ to Ni^2+^. Adopted with permission from Ref. [83].

Escote et al. have proposed another non-enzymatic electrochemical biosensor comprised of a NdNiO_3_nanotubes-graphene oxide electrode (GO/NNO) for sensitive determination of AA (Figure 16) [83]. Herein, two GO/NNO have been fabricated with two different external diameters of 20 nm (NNO20) and 100 nm (NNO100), respectively. Based on the pore size and the ratio of Ni^3+^/Ni^2+^, the sensitivities of the electrodes are varied. It has been observed that in NNO20, trivalent nickel (Ni^3+^) was more stabilized, while in NNO100, divalent nickel (Ni^2+^) was more stabilized. Before interaction of NNO20 with AA, there are a number of active sites available due to the presence of a partially filled d-orbital of Ni^3+^ (t_2g_^6^ e_g_^1^). Then, the protons formed after the oxidation of AA underwent H-bonding interaction with the oxygen anions of NNO. In this situation, the extra electron is located in the Ni_3d_-O_2p_ hybridized orbital, which led to the oxidation of AA to DHAA with the corresponding reduction of Ni^3+^ to Ni^2+^ (t_2g_^6^ e_g_^2^) of NNO. Although the presence of Ni^3+^ is more beneficial due to the presence of the singly-occupied orbital, oxygen deficiency is an imperative parameter of the electrochemical sensing phenomenon due to the presence of abundant active sites. Therefore, it has been observed from chronoamperometric measurement that the electrochemical sensitivity for AA detection is greatly improved in the GO/NNO100 system (sensitivity = 0.031 µAµM^−1^cm^−2^, LOD = 3.8 µM) over the GO/NNO20 system (LOD = 11.1 µM, sensitivity = 0.023 µAµM^−1^cm^−2^). The sensitivity of the material has been exploited towards sensitive detection of AA from synthetic sweat samples with a recovery time period of ~33 S.

**Figure 17:**
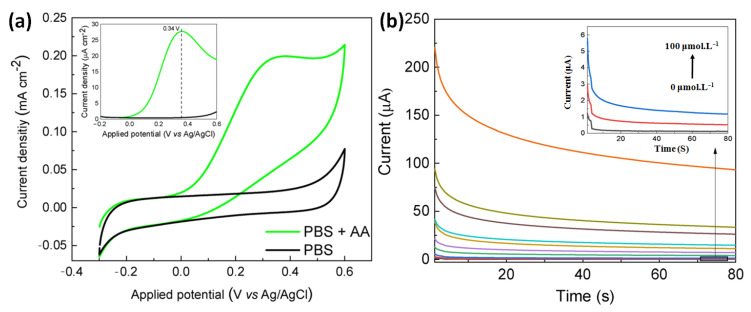
(**a**) Cyclic voltammogram of PFA/GO-3.88 W electrode in the presence of AA in PBS buffer media (inset: the corresponding differential pulse voltammogram data, scan rate of 50 mVs^−1^, (**b**) chronoamperometric data of the electrode in the presence of increasing concentrations of AA from 0–5000 µM. Adopted with permission from Ref. [84].

Pope and Carreno et al. have developed another non-enzymatic sensor by laser-induced (at the power of 3.88 W) carbonization of waste biomass-derived poly (furfuryl alcohol)/graphene oxide (PFA/GO-3.88 W) electrodes for selective detection of AA [84]. The produced electrodes provided a unique combination of porous morphology, a large amount of sp^2^ carbon content, edge plane defects, etc., for efficient electrocatalytic activity. Initially, the electrochemical detection of AA proceeded via the adsorption of AA onto the electrode. Then, water led to hydrolysis of AA to produce dehydroascorbic acid, losing two electrons, which was responsible for the variation of current detected electrochemically via CV, DPV and chronoamperometry (Figure 17). Moreover, the generation of a new irreversible peak at 0.34 validates the oxidation of carbonyl functionality on the furan ring. Increased electronic states density, i.e., carboxylic functionality at the basal plane of the electrodes, facilitated the electrocatalytic oxidation of AA. Additionally, aromaticity led to the facile π-π stacking interaction of AA onto the electrodes followed by charge transfer. PFA/GO-3.88 W was also useful for potential detection of AA from human sweat with improved sensitivity (0.0485 µA µM^−1^ cm^−2^) and a very low LOD of 1.33 µM cm^2^.

**Figure 18:**
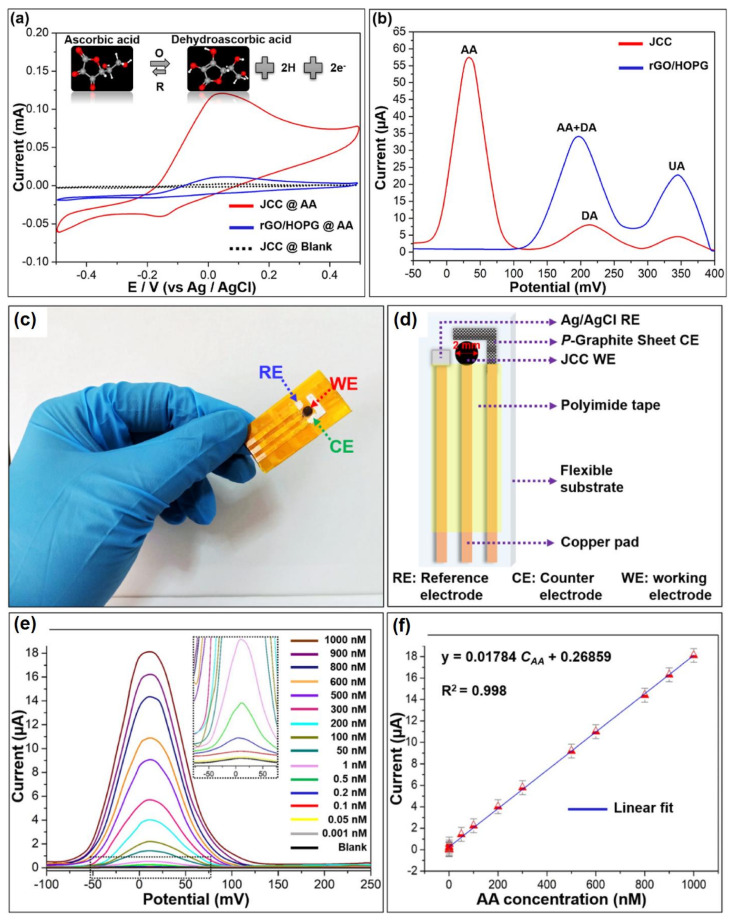
(**a**) Cyclic voltammograms of the rGO/HOPG and JCC electrodes in absence/presence of 0.1 mM AA at a scan rate of 100 mVs^−1^, (**b**) DPV results in presence of the 0.05 mM AA, 0.5 mM dopamine (DA), and 1 mM uric acid (UA), (**c**) photographic image and (**d**) schematic diagram of the fabricated portable point-of-care device, (**e**) DPV curves in presence of increasing concentration of AA and (**f**) the corresponding calibration curve. Adopted with permission from Ref. [85].

Wu et al. have developed a Janus carbon nanocomposite (JCC) from melamine, reduced graphene oxide (rGO), and highly ordered pyrolytic graphite (HOPG) for sensitive determination of AA [85]. From the cyclic voltammogram data, it has been observed that only in the presence of AA on the JCC electrode was a significant anodic peak obtained at 32 mV. Along with this, a current response of 122 µA was noticed, which corresponds to irreversible oxidation of AA (Figure 18a). To obtain the precise information, DPV studies were carried out, which unambiguously exhibited the paramount selectivity of AA even in the presence of its potentially interfering analytes, such as dopamine (DA), uric acid (UA), etc. (Figure 18b). This has also been verified by theoretical DFT studies, which indicate greater stability as well as shorter H-bonding distance in AA adsorption. To explore the flexibility and commercial use, a handy point-of-care device was fabricated from real-time AA detection in sweat specimens (Figure 18c–f).

### 4.5. Ethanol @ aMajor Biomarker for Drunk Driving

Alcohol, i.e., the diluted form of the absolute “ethyl alcohol” or “ethanol” is one of the most significant products of global addiction. Alcohol consumption is directly or indirectly connected with various chronic diseases, such as liver cirrhosis, cardiovascular diseases and even cancer [86,87]. According to WHO, alcohol consumption leads to the death of almost three million people annually worldwide, along with ~5.1% of Disability-Adjusted Life Years (DALYs) of the world [88]. This trend may have a huge impact on the young generation (under 25), who are most addicted to binge drinking on a regular basis [89]. Moreover, drunk driving may also be responsible for road accidents [90]. Due to various socio-economic practices, alcohol consumption is also growing day by day in developing countries, such as India [91], which leads to a yearly loss of 1.45% on average of the gross domestic product (GDP) of the Indian economy [92]. Thus, chronic alcohol consumption not only leads to physical burden, but also financial crunch. Transdermal sweat content is directly correlated to blood alcohol level. Therefore, the monitoring of alcohol consumption from sweat by means of a portable device inevitably reveals a person’s intoxicated state.

Alcohol consumption is also related to type 2 diabetes, in which the level of glucose inside the body is increased. In this background, recently, Prasad et al. have reported a lancet-free, label-free, flexible electrochemical ethanol and glucose sensor to establish a direct correlation of alcohol consumption with glucose level enhancement in the body [93]. The electrochemical sensor was comprised of zinc oxide thin films-conjugated nanoporous flexible electrodes followed by immobilization with alcohol oxidase and glucose oxidase enzyme. Herein, the biomolecular interaction followed by catalytic oxidation of alcohol led to the corresponding modulation of the capacitive reactance, which was monitored via variation of the imaginary impedance (Z_imag_), characterized by EIS. The percentage variation of Z_imag_ for 0.01 mg/dL ethanol in 0.1×PBS at 50 Hz and 500 Hz was discovered to be ~8.5%, while at the alike condition, the percentage variation of Z_imag_ for 200 mg/dL ethanol was noticed to be ~30–35%. It has been observed that with an increasing pH of the synthetic sweat from 4 to 8, the percentage variation of Z_imag_ was also increased. Accordingly, the detection threshold of ethanol in the synthetic sweat was revealed to be 0.01 mg/dL in the pH range of 4–8. The determination of glucose and ethanol in sweat has been successfully carried out parallelly by their developed sensor, which is highly comparable with the commercially available analyzers.

**Figure 19:**
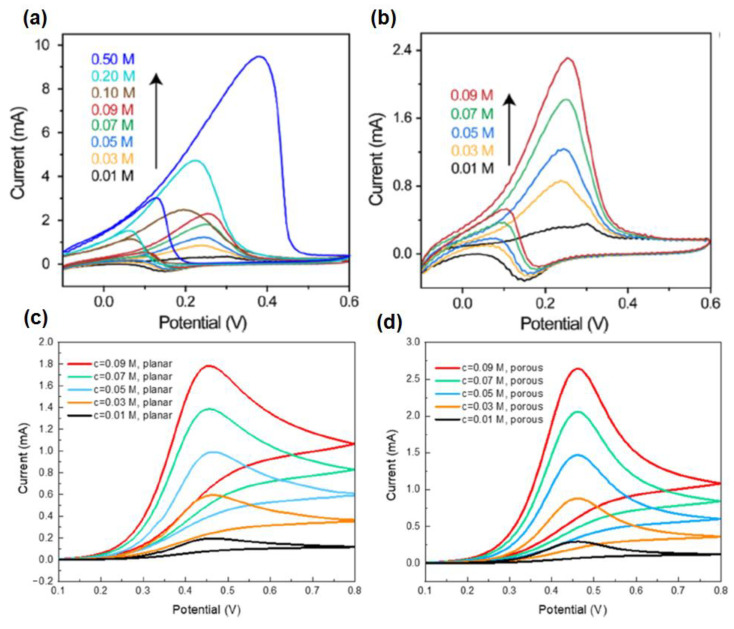
Current response of Au NW-gel in presence of the ethanol concentration in a range of (**a**) 0.01–0.50 M and (**b**) 0.01–0.09 M; and cyclic voltammogram simulation response on (**c**) planar and (**d**) porous electrode surface.

Yang et al. have reported a 3D gold nanowire aerogel (Au NW-gel) based bioelectrode for monitoring ethanol non-invasively in simulated sweat by cyclic voltammetry [94]. Herein, Au NW-gel was chosen as a sensor material, as it has high gold content and ultralow density, which reduces the internal as well as mass transfer resistance. It has been observed that with increasing the concentration of ethanol, the current response is increased in the scanning potential window of −0.1 to 0.6 V (vs. Ag/AgCl) at the scan rate of 20 mV s^−1^. This enhancement of the current response may be ascribed to the increased rate of oxidation of ethanol to acetaldehyde, catalyzed by Au NW-gel. The sensor can detect ethanol in a broad concentration range of 0.01–0.5 M (Figure 19a,b). For illustrating electrochemical reaction kinetics and performance of mass transfer, cyclic voltammogram simulation studies were carried out both in a planar as well as a porous electrode surface. This exhibits that the response on the porous electrode is much greater than that of the planar electrode surface due to having a milli-amperes (mA) range of current on the porous surface along with the smaller size of the electrode with respect to that of the external electrolyte (Figure 19c,d).

**Figure 20:**
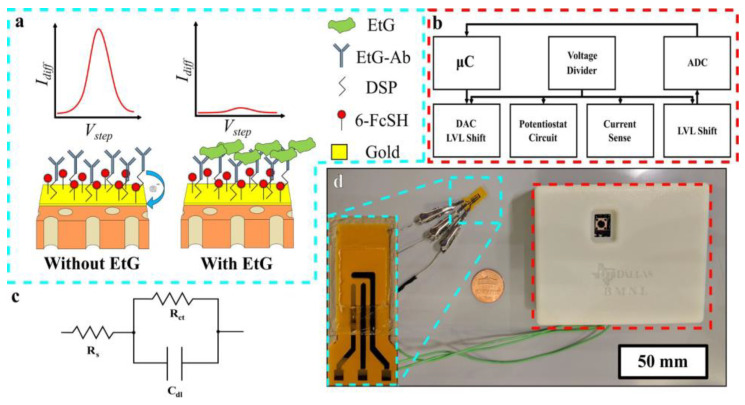
(**a**) Schematic illustration of the biosensor in the absence (left) and presence (right) of EtG, (**b**) block diagram of the major circuit elements of the constructed compact reader, (**c**) modified Randles circuit, and (**d**) pictorial representation of the portable EtG biosensor, with a close-up view of the sensor (inset).

However, the major drawback of the sweat or transdermal alcohol sensor is that the sensitivity window of the corresponding sensors is very short due to the volatile nature of alcohol. Therefore, to curtail the loopholes, Prasad et al. have reported the sensing of ethyl glucuronide (EtG) [95], which is generated by the glucuronidation of alcohol and is capable of measuring it in sweat over 24 h after light drinking, which may last even up to 4 days after heavy drinking [96]. In human sweat, the level of EtG lies in the range of 1.7–103.0 µg/L after consumption 38.0–154.6 g of alcohol. However, there will not be any trace of EtG if ethanol is not consumed. Herein, a gold electrode was surface functionalized with monoclonal antibodies in parallel with dithiobis (succinimidylproprionate) (DSP) and a charge transfer compound, 6-ferrocenyl-1-hexanethiol (6-FcSH) for selective detection of EtG from human sweat via a DC-based sensing mechanism using SWV as the transduction method. In the absence of EtG, the charge transfer process of 6-FcSH was restricted, which resulted in a stronger current response. However, in absence of EtG, the charge transfer process of 6-FcSH is no longer inhibited due to molecular crowding. This manifested a smaller current response during SWV. Ultimately, to make the sensor as a portable device, the sensor was fabricated on a nanoporous flexible polyimide membrane (Figure 20).

**Table 2:** Detection of sweat metabolites from sweat specimen by electrochemical sensors integrated with nanomaterials.

Targeted Sweat Metabolites	Nanosensors	Detection Principle	Sensitivity	Detection Limit	Linear Range	Ref.
Glucose	Hydrogel-based microneedle system	Alteration in the heights and swelling ratios of microneedles and potentiostat	-	-	8–22 mM	[60]
	Enokitake mushroom-like standing gold nanowires	Chronoamperometry	23.72 µA·mM^−1^·cm^−2^	10 µM	0–800 µM	[61]
	Gold nanostructure	Chronoamperometry CV	-	7 µM	25 to 250 µM	[62]
	GOx/PtNP/acetic acid treated LIG	Chronoamperometry CV	4.622 µA/mM	300 nM	0.0003–2.1 mM	[63]
	Nickel phosphate nano flakes	Chronoamperometry CV	24.39 mA·mM^−1^·cm^−2^	97 nM	10–1000 µM	[64]
	TiO_2_ nanotube arrays@Ti mesh	CV	-	0.0361 mM	0.02–0.7 mM	[73]
Urea/Uric acid	alpha nickel hydroxide nanoparticles	Chronoamperometry CV	2.5 ± 0.08 µA	2.28 × 10^−8^ M	-	[67]
	TiO_2_ nanotube arrays@Ti mesh	CV	-	2.0675 mM	1–70 mM	[73]
Lactic acid	AuPtNPs functionalized MoS_2_ nanosheet	CV EIS SWV	-	0.00033 mM	0.005–3 mM	[70]
	Zinc oxide nanoflakes	CV EIS	11.76 µA/decade/cm^2^	1.26 nM	10 pM–20 mM	[71]
	Graphene oxide (GO) nanosheets	EIS	-	1 mM	4–80 mM	[72]
	TiO_2_ nanotube arrays@Ti mesh	CV	-	0.0131 mM	0.8–7 mM	[73]
	Gold nanopine needles	Chronoamperometry CV	-	54 µM	-	[62]
Ascorbic acid	Carbon quantum dot electrode based enzymatic sensor	Amperometry	2.02 nA µM^−1^	12 nM	1–400 µM	[77]
	Modified gold microelectrodes with trapped CuO nanoparticles	Amperometry CV	0.103 V log (µM)^−1^ of peak shift	1.97 µM	10–150 µM	[81]
	Nanorod of conductive Ni-MOF	CV	0.814 µA µM^−1^ cm^−2^	1 µM	2–200 µM	[82]
	NdNiO_3_ nanotubes supported on GO flexible electrodes	CV chronomperometry	0.031 µA µM^−1^ cm^−2^	3.8 µM	30–1100 µM	[83]
	Laser induced carbonization of graphene oxide filled biomass-derived polymer poly(furfuryl alcohol) (PFA/GO) based non-enzymatic sensor	CV DPV chronomperometry	0.03748 µA µM^−1^ cm^−2^	1.0 µM	50–5000 µM	[84]
	Janus carbon nanocomposite	CV EIS DPV	-	47 pM	10^−12^ M–10^−6^ M.	[85]
Ethanol	Zinc oxide thin film electrodes, surface functionalized with alcohol oxidase enzyme	EIS	0.2 ± 0.02 µA/mM	0.01 mg/dL	0.01–200 mg/dL	[93]
	Gold nanowire aerogel (Au NW-gel)	CV EIS	-	-	0.01–0.5 M	[94]
Ethanol metabolite, ethyl glucuronide (EtG)	Gold electrode, surface functionalized with monoclonal antibodies and thiolated charge transfer molecule	SWV	-	0.1 µg/L	0.1–100 µg/L	[95]

## 5. Nanomaterials Integrated Wearable Electrochemical Sensors@ Need of the Hour for Personalized Health Monitoring

**Figure 21:**
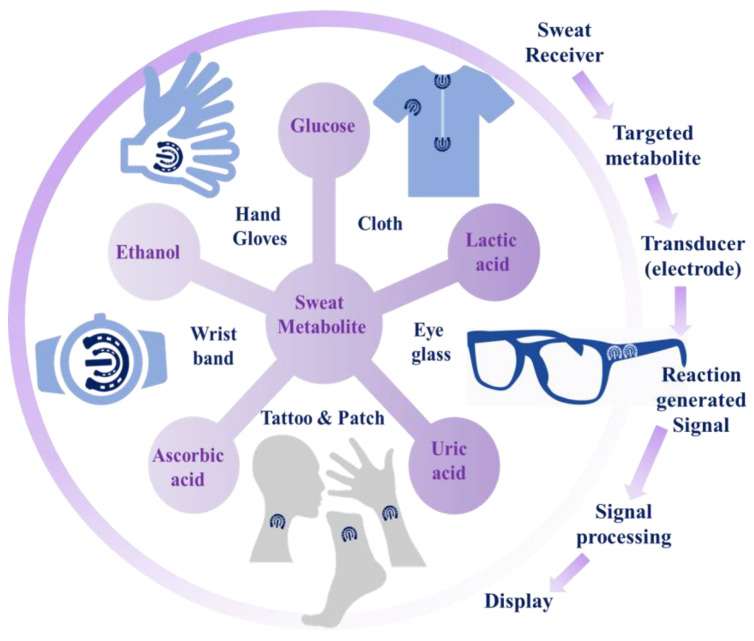
Schematic working procedure of wearable sweat metabolite sensors.

Sweat comprises an affluence of biochemical information as a potent indicator of the body’s profound biomolecular state. Although sweat exhibits its own limitations in the measurement of biomarker concentrations, it is advantageous over other biofluids that have uplifted it as a preeminent contender in the realm of wearable technology. In most cases, sweat is allowed to generate naturally by workouts or exposure to humid environmental conditions. However, owing to several limitations associated with the aforementioned solutions for sweat generation, localized production of sweat via iontophoretic transdermal charged drug (pilocarpine, carbachol, acetylcholine, etc.) delivery has been found to be more appropriate. Wearable devices adopt this position-sensing mechanism among combined electrodes covered by an iontophoretic stimulant gel. Wearable sensors, positioned adjacent to the perspiration generation site, would permit prompt recognition before the biodegradation of the analytes [97,98,99]. Approximately all existing metabolite-detecting wearable sensors rely on a particular enzyme for selective detection of the targeted analyte. Furthermore, sweat metabolites measuring wearable sensors are non-invasive and can be implemented on-demand at apposite sites on the body for continuous monitoring (Table 3). The emerging arena of wearable sensors aims to provide individuals with vision into the subtleties of their individual physiology. The durable vision upholds sensors that can be combined into wearable setups such eyeglasses, wristbands, patches, clothing, or tattoos for continuous investigation of an assortment of body indicators. Depending on the biological information produced in the body of healthy and sick states, these sensors will permit personalities to monitor the individual’s health status in an inexpensive way without trained personnel (Figure 21).

### 5.1. Wearable Glasses Sensor

Eyeglasses are extensively used on numerous junctures as they offer vision adjustment and substantial defense against eye wounds. The arms of the eyeglass frame are adjacent to the skin of the head and preserve an impression against the skin, which establishes a platform on which the miniaturized biosensor with protection can be mounted, avoiding deformational interference [100,101].

**Figure 22:**
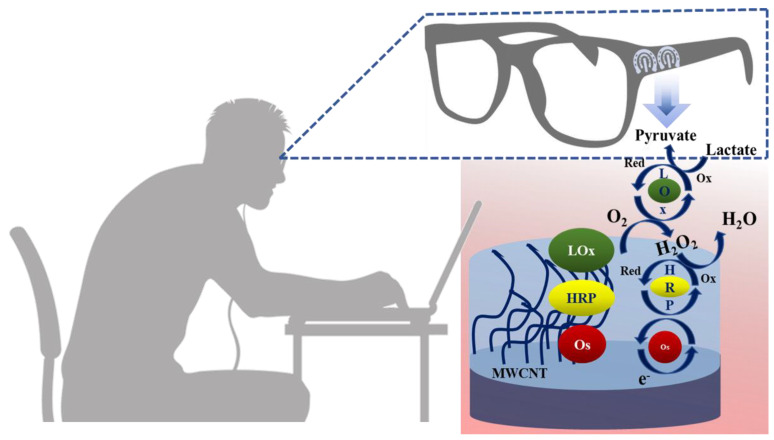
Schematic presentation of biosensor integrated with glasses for lactate monitoring in human sweat, symbols: LOx (ox), LOx (red), HRP (ox) and HRP (red) are the oxidized and reduced forms of the aforementioned enzymes, respectively.

Recently, Zhang et al. have developed an enzymatic biosensor embedded in eyeglasses for non-invasive monitoring of lactate in perspiration (Figure 22). Herein, the Os-complex acts as an electro-mediator between the enzyme and the electrode. A gel membrane of lactate oxidase was impregnated on the electrode to form the biosensor. L-lactate was oxidized in the attendance of lactate oxidase (LOx) to pyruvate and H_2_O_2_. An immobilized Horseradish peroxidase enzyme (HRP) was oxidized by hydrogen peroxide to produce H_2_O, which changed HRP from a reduced to an oxidized form. Again, the oxidized form of HRP was reduced by divalent osmium (Os(II))-centered redox polymer. Chemical selectivity was assessed by the addition of interferents such as glucose, ascorbic acid, and uric acid. The amperometric outcomes of the biosensor indicated the concentration-dependent response of the sensor in varying concentrations of sweat lactate. The mounting of the aforementioned biosensor on eyeglasses can be simply worn, and the real-time monitoring of lactate levels can be measured without disruption since the biosensor remains in touch with the skin. Such application-oriented instantaneous monitoring of lactate through eyeglass-bound biosensors has real importance [101].

### 5.2. Wearable Gloves Sensor

Gloves, disposable materials worn on the user’s hand [102], are potential candidates in the domain of biosensing applications by integrating the electrochemical sensors into the gloves [103]. The sweat glands’ densities on the backside of the palm or finger, i.e., the dorsal side, are very high. This makes them a potential target area for sweat collection in comparison to the other body parts generally used for sweat analysis by wearable biosensors. Moreover, with the creation of an enclosed environment with low evaporation of the secreted sweat within gloves, the gloves-based biosensor can efficiently collect enough sweat from the dorsal side of the hand for monitoring health conditions. Therefore, integration of the bio-electronics with a wearable platform such as gloves can be immensely helpful for regular monitoring of self-health status. Recently, wearable nitrile-based gloves are being widely used as a potential substrate for monitoring common sweat metabolites, e.g., lactate, uric acid, ascorbic acid, ethanol, drug metabolites, etc. [104,105,106].

**Figure 23:**
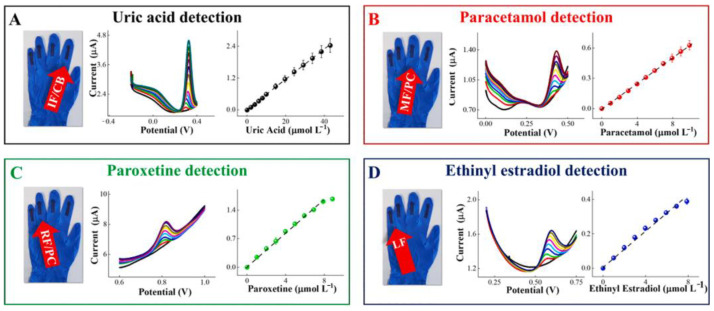
Glove-embedded wearable electrochemical sensors containing a full electrochemical set up in each finger: (**A**) IF/CB for uric acid detection, (**B**) MF/PC for paracetamol detection, (**C**) RF/PC for paroxetine detection and (**D**) LF/pretreated for ethinyl estradiol detection. Adopted with permission from Ref. [104].

Raymundo-Pereira et al. have reported a glove-embedded wearable non-enzymatic sensor for simultaneous electrochemical sensing of important sweat metabolites, uric acid along with three drug metabolites, paracetamol, paroxetine and ethinylestradiol (EE2) [104]. For this purpose, four sensors were printed on the four fingers (index (IF), middle (MF), ring (RF) and little fingers (LF)) of a nitrile glove for the simultaneous detection of four analytes via DPV technique. Carbonaceous nanomaterials were adopted to functionalize the sensing layers for improving the sensitivity of the electrochemical sensors. The working electrode of IF was modified by carbon black (CB), while MF and RF were modified with Printex 6L carbon (PC) and LF was electrochemically pretreated with 0.1 M H_2_SO_4_ medium to obtain the corresponding MF/PC, RF/PC, LF/pretreated and IF/CB of the glove-embedded sensors to simultaneously detect uric acid, paracetamol, paroxetine and ethinylestradiol, respectively, from sweat via electro-oxidation of each analyte to their oxidized forms (Figure 23). Stretching and bending deformation studies with the electrochemical fingerprints have been performed for 50 repetitive cycles, which indicates the immense mechanical stability of the glove-embedded sensors. Thus, by attaching the fingertips of the glove to the sweaty skin or by dipping the fingertips in the sample specimen, real-time non-invasive analysis of the targeted analytes is possible.

**Figure 24:**
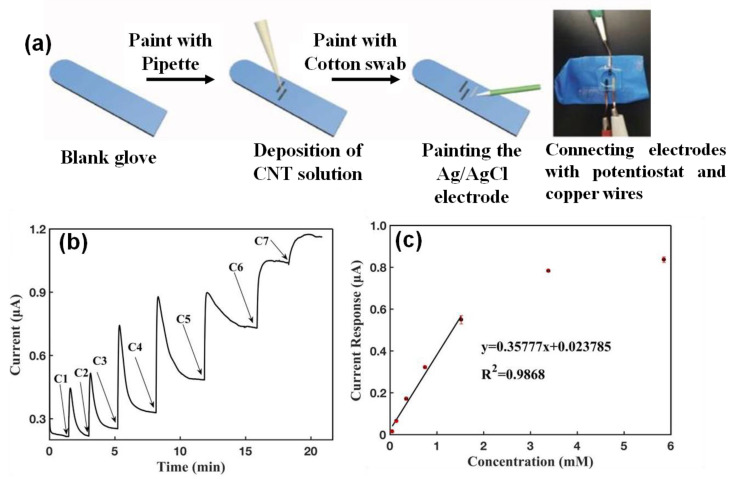
(**a**) Schematic illustration of the carbon nanotube- (CNT) and/or Ag/AgCl inks-based wearable enzymatic amperometric sensor and related connection methods, (**b**) current-versus-time response curve upon addition of lactate on the glove sensor and (**c**) the corresponding calibration curve.

Cui et al. have reported carbon nanotube- (CNT) and/or Ag/AgCl inks-based wearable enzymatic amperometric sensors, coated onto nitrile gloves functionalized with lactate oxidase for sweat lactate monitoring [105]. Herein, lactate oxidase catalyzed the conversion of lactate to H_2_O_2_. At 0.6 V potential at working electrode vs. reference electrode, signal response could be acquired via oxidation of H_2_O_2_. It has been observed that, by increasing the concentration of H_2_O_2_, the current signal response was also increased, which could indirectly measure the concentration of the targeted analytes (Figure 24). Herein, three sensor configurations have been adopted. In the first two-electrode configuration, CNT-based electrodes have been employed as working and reference/counter electrodes. In the second two-electrode configuration, a CNT-based electrode has been used as the working electrode, while a Ag/AgCl electrode was used as the reference/counter electrode. On the other hand, in the third, three-electrode configuration, two CNT electrodes were used as both working and counter electrodes, while a Ag/AgCl electrode was employed as a reference electrode. It was discovered from the electrochemical experiments that the second and third configurations exhibited greater sensitivities than the first configuration. The performance of the CNT-based electrode was found to be excellent as a working or counter electrode, but not that very effective as a reference electrode, as the working electrode potential would be unstable in such a case. Moreover, the present sensor can distinguish concentrations of lactate at different exercise conditions, e.g., running, cycling, and jogging.

**Figure 25:**
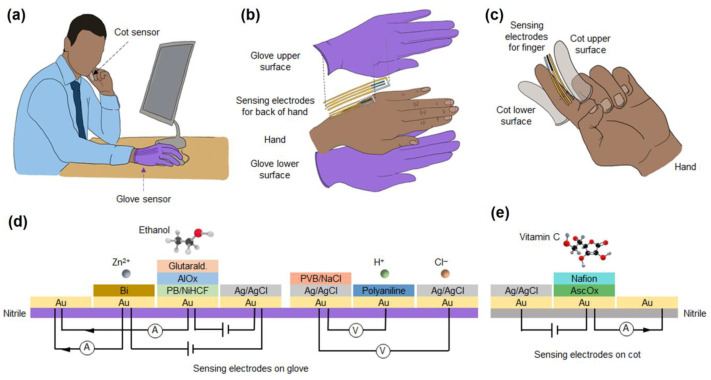

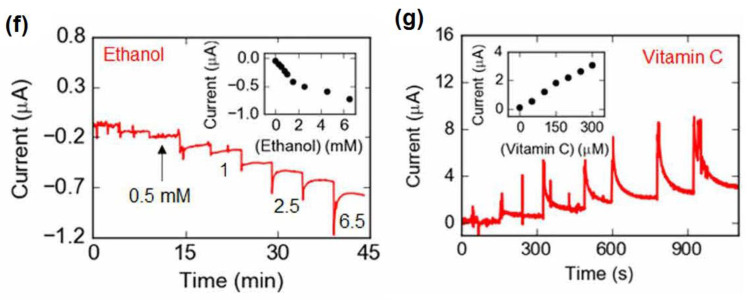
(**a**) Glove-based electrochemical sensor for monitoring the targeted analytes during routine activity, (**b**) fabrication of electrochemical sensors on the versatile glove positions, e.g., at the back of the hand for obtaining sufficient amount of sweat, (**c**) functionalized finger cots for monitoring sweat vitamin C, (**d**) schematic diagram of the cross-sectional sensing layer for the recognition of sweat metabolites, (**e**) nitrile cot functionalized with vitamin C sensor, calibration curves for (**f**) ethanol and (**g**) vitamin C in buffer solutions at different concentration.

Bariya and Li et al. have developed a glove-based xenobiotic sensor for real time electrochemical detection of sweat ethanol and vitamin C (ascorbic acid), placing the sensors inside powder-free nitrile gloves in contact with the back of the hand and finger cots [106]. The gloves were patterned with an alcohol sensor, which was comprised of gold nanodendrites as working an electrode functionalized with alcohol oxidase enzyme, a Ag/AgCl reference electrode and a gold counter electrode. On the other hand, the nitrile finger cots were patterned with ascorbate oxidase for analyzing the sweat vitamin C (Figure 25). The electrode sensors ended at the wrist, which was connected to a potentiostat for signal extraction. The chronoamperometric calibration of ethanol and vitamin C was carried out 0 and 0.2 V, respectively. The on-body trial of the sensors was carried out by wearing the gloves or cots for 30 min to obtain a sufficient amount of sweat to acquire a reliable outcome.

### 5.3. Wearable Patch Sensor

In the quest for noteworthy requirements for incessant monitoring of disease biomarkers from sweat with real-time non-invasive sensing pathways, wearable skin patch sensors have been developed. The epidermis is the outmost skin layer, usually forming the main barrier of diffusion for nearly all environmental compounds. Moreover, acquiring data about sweat metabolite concentrations from skin is more difficult due to the lack of strategies for fixing the miniaturized electronic part on a thin substrate, which can collect sweat-related information and analyze it accordingly. Additionally, due to the soft, heterogeneous, viscoelastic, anisotropic, and curvilinear nature of human tissue, it experiences consistent multiple-axes distortions. Consequently, for skin-adherable patch-type sensors to be easily tangled onto the ununiform surface of human skin, they should have mechanical properties similar to human skin. Wearable skin patch-based bio-sensors are low in weight, engraved with miniaturized electronics intended for incessant amalgamation with the body and require less effort. Their sensing principle and quantitative recognition approach depend on the combined electrodes, which do not require the calibration step. Most of the non-invasive sensors are enzymatic sensors, while few are non-enzymatic. Much effort has been devoted to the fabrication and functionalization of the requisite sensors for obtaining improved sensitivity [107].

**Figure 26:**
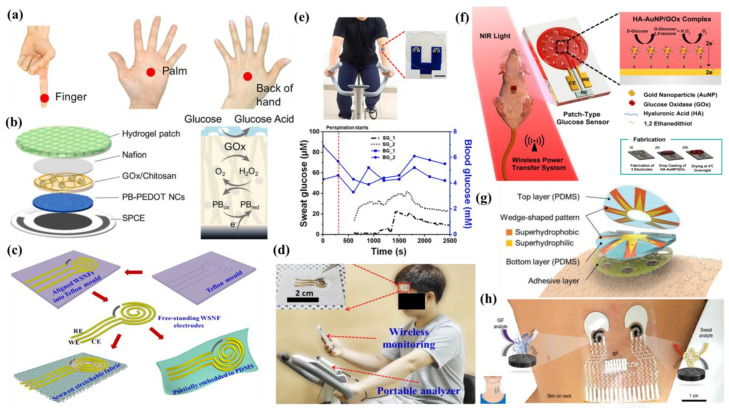
(**a**) Accessible positions on the finger, palm and back of the hand for sweat collection; (**b**) schematic of the multiple layers in the enzymatic PB-PEDOT NC electrode of the sweat glucose sensor and the GOx working mechanism with the PB probe;(**c**)schematic diagram of the fabrication process for free-standing WSNF electrodes (CE, WE, and RE) partially embedded on a PDMS substrate or sewn onto stretchable fabric to produce a stretchable WSNF glucose sensor; (**d**) WSNF glucose sensor sewn onto a stretchable fabric (inset) and attached to the forehead of a human body, adopted with permission from Ref. [109]; (**e**) photographs of the sweat patch worn on the arm of the subject during cycling with an enlarged photo showing the device. Scale bar: 1 cm and continuous measurement of sweat glucose level before (short black dash-dot: SG_1) and after taking meal (short black dot: SG_2). Periodic measurement of blood glucose every 5 min pre- (square blue dot: BG_1) and post-meal (circle blue dot: BG_2), adopted with permission from Ref. [110]; (**f**) schematic illustration for the wireless patch-type glucose sensor system using hyaluronate (HA)–gold nanoparticle (AuNP)/glucose oxidase (GOx) complex and the glucose-sensing mechanism for the non-invasive real-time monitoring of glucose in sweat, adopted with permission from Ref. [111]; (**g**) schematic illustration of sweat-collecting patch, adopted with permission from Ref. [120]; and (**h**) illustrations of the placement of the sensor and the enzymatic chemical sensors for ISF and sweat.

Lin et al. have invented a non-invasive sweat glucose sensor using hydrogel patches coupled with an electrochemical glucose sensor for monitoring sweat glucose levels (Figure 26a,b) [108]. A hydrogel-based patch is attached to the skin as a sweat receiver. The perspiration transports glucose-like sweat metabolites on the surface of the skin. The interface enables the diffusion of metabolites from sweat to hydrogels, and the requisite analytes are collected in the patches. The hydrogel reservoir then transfers the contents to the multilayered sweat glucose sensor with a nanostructure film, which shows electrochemical sensitivity. The sensor layers were constructed as, from bottom to top, conducting layers of Prussian blue (PB) and poly (3,4-ethylenedioxythiophene) nanocomposites, a GOx immobilization layer and a layer of Nafion. In the presence of glucose, the GOx layer generates H_2_O_2_, which rapidly catalyzed the reduction of PB at a low overpotential. The response signal is monitored at different sweat gland enriched positions of the body by the developed glucose sensor.

Toi et al. have developed a self-monitoring patch biosensor that can detect sweat glucose (Figure 26c,d) [109]. Herein, they have fabricated a non-enzymatic, electrochemical sensor-based patch that is stretchable, and nanohybrid fiber (WSNF) and Au nano wrinkles have enclosed the reduced graphene oxide/polyurethane (rGO/PU) composite fiber. The WSNF exhibited high electrocatalytic activity due to the collaborative effects between the Au nano-wrinkles and the oxygen-comprising functional groups on the rGO, which enabled the dehydrogenation step in glucose oxidation. The stretchable WSNF offers excellent sensitivity and a low detection limit in neutral pH.

Sun et al. have developed non-invasive microfluidic patch, which is based on a “cut-and-paste (CAP)” methodic pattern by transforming the polydimethylsiloxane (PDMS) and polyethylene terephthalate (PET) film (Figure 26e) [110]. The “bear like” patch, comprised of four polymer-based layers, was used for sweat glucose and lactate monitoring purposes. The performance of the sensor was externally checked by the gradual addition of glucose and lactate solutions. The glucose biosensor exhibited a wide linear range and a promising sensitivity with a LOD of 7.34 µM. Similarly, the lactate sensor exhibited a wide linear response range with a LOD of 1.24 mM. The glucose sensor patch exhibited an improved current response at a flow rate of 20 µL min^−1^ and decreased progressively as the flow rate remained constant after the increment. Additionally, the lactate sensing patch showed an extremely symmetrical I-T curve, exhibiting a linear relation among the current response and a flow rate lower than 50 µL min^−1^ as the flow rate increased from 0 to 50 µL min^−1^. This indicated that alterations in the sweat rate would deviate from the actual concentration value for metabolites under dynamic circumstances.

Zhang et al. have reported a wearable electrochemical biosensor based on Ag Nanowires (AgNWs) and molecularly imprinted polymers (MIPs) on the SPE for the non-invasive detection of lactate in sweat (Figure 26f). The MIPs were fabricated by electro-polymerization of 3-aminophenulboronic acid (3-APBA) with an imprinted lactate molecule on the AgNWs-coated working electrode. The MIPs were modified with an electrophilic para-substitution by the amino group. Furthermore, the boronic acid residue of 3-APBA helps in the formation of a stable adduct with lactate at low pH. Owing to the allocation of the negative charge on the boron atom, poly (3-APBA) acts as an electron donor group. The MIPs-AgNWs biosensor showed high sensitivity and specificity for the recognition of lactate from 10^−6^ M to 0.1 M, with a LOD of 0.22 µM. In addition, the flexible electrodes also showed stable electrochemical responses [111].

Kil et al. have fabricated a wearable patch-type self-charging supercapacitor for measuring sweat glucose. The glucose oxidase enzyme covered on the surface of the microneedle-type sensor detected glucose in the interstitial fluids of the human body. The electrons generated by the glucose oxidation process start the self-powered system, in which charging commences with the generation of potential differences in supercapacitor electrodes. The CV curves indicated that only in presence of glucose does a redox reaction occur, with the signature peak appearing at approximately −0.45 V. In an 11 mM glucose solution, the self-powered supercapacitor (SPSC) exhibited a power density of 0.62 mW/cm^2^. Therefore, the self-powered glucose sensor has efficiently discriminated normal, prediabetic, and diabetic levels in a laboratory skin model [112].

Shu and Hu et al. have developed a wearable sensor based on Au/PDMS film coated with a Ni–Co MOF nanosheet with high electrocatalytic activity for the monitoring of sweat glucose [113]. AuNPs were deposited onto the PDMS film to make the electrode stretchable and highly conductive in nature. The Ni–Co MOF nanosheet was then deposited to further modify the working electrode for the electrocatalytic oxidation of glucose. A sweat-adsorbent cloth was used to cover the working area of the sensor for collecting the sweat from arm skin. The reversible redox behavior of the Au/PDMS film-coated Ni–Co MOF nanosheet made it suitable for unlimited molecule or electron transfer. The sensitivity of the sensor was checked by varying the metal ratio, and it has been observed that a nickel to cobalt ratio of 1:5 was optimal for the electrocatalytic oxidation of glucose. From cyclic voltammogram and amperometric data, it has been observed that with enhancement of the concentration of glucose, the oxidation current value gradually increased due to the oxidation of glucose to gluconolactone with simultaneous reduction of Co(IV) and Ni(III) to Co(II) and Ni(II), respectively. The response is largely comparable with the commercially available glucose meter.

The same group has again reported that Ni−Co metal−organic framework/Ag/reduced graphene oxide/polyurethane (Ni−Co MOF/Ag/rGO/PU) fiber-derived stretchable wearable sensor for electrochemical sensing of glucose from sweat as fiber-based wearable patch sensors are always advantageous due to an excellent wearing experience [114]. Herein, a waterproof stretchable band was fixed on a PDMS substrate for on-body sweat analysis. From the cyclic voltammogram and amperometric response, it has been observed that with increasing the concentration of glucose, the current response was also enhanced due to promising electrocatalytic activity of Ni−Co MOF via oxidation of glucose to gluconolactone with the simultaneous reduction of Co(IV) and Ni(III) to Co(II) and Ni(II), respectively. On-body glucose detection performance of the developed electrochemical sensor was also examined by attaching the sensor patch on the volunteer’s arm after collecting sweat with a sweat-absorbent cloth.

Kinnamon and Sankhala et al. have developed a wearable electrochemical sensor (AWARE) for real-time monitoring of alcohol consumption via tracking ethyl glucuronide (EtG) in sweat specimen [115]. Herein, non-faradic electrochemical impedance spectroscopy and antibody-based immunoassay have been adopted for establishing a relationship between ethyl glucuronide, present in a sweat specimen after having a certain number of drinks in a given time period. The electrode was made with a conductive gold electrode over a conductive layer (~125 nm) of ZnO thin film, which was then functionalized with anti-EtG monoclonal antibodies. The generated sweat from the healthy volunteers was collected by a PharmChek patch. Upon the interaction of EtG with anEtG-specific antibody, there was modulation in the electrical double layer (EDL), which resulted in the variation of impedance of the electrochemical sensor. The sensor response could be translated via a Bluetooth or USB connection. The sensitivity of the sensor was comparable with that of the benchtop potentiostat.

Cai and Ye et al. have reported a flexible hydrogel-paper patch (HPP) integrated with poly (3,4-ethylenedioxythiophene): poly (4-styrenesulfonate) (PEDOT:PSS) and Pt nanoparticles-based enzymatic sensor for on-time scrutinizing of sweat glucose [116]. Herein, popcorn-like Pt nanoparticles were exploited for amperometric H_2_O_2_ sensing, which was generated via electro-oxidation of glucose in the presence of a glucose oxidase enzyme. The sensitivity of the sensor was investigated with the sweat samples of healthy volunteers after fitness cycling, from which the data could be wirelessly transmitted to a smartphone via Bluetooth. Thus, the present work of glucose sensing would be helpful for the evaluation of the fatigue levels of athletes.

Kim et al. reported a non-invasive sensor for measuring body glucose levels. The sensor was comprised of a hyaluronate-gold nanoparticle/glucose oxidase (HA-AuNP/GOx) complex and an ultra-low power application-specific integrated circuit (ASIC) chip for the non-invasive and robust wireless patch [117]. The complex was equipped with the facile conjugation of thiolated HA to AuNPs and the following physical binding of GOx. The as-generated electrons from the reaction of glucose and GOx flow from the working electrode to the counter electrode, resulting in the current change. In this way, from the gradient of current changes compare to that of the reference electrode, the glucose concentration can be measured. The wireless glucose sensor exhibited prompt response (5 s), large sensitivity and low detection limit of 0.5 mg/dL. Herein, the glucose concentration levels were compared with a commercially-available blood glucometer and the wireless patch-type glucose sensor as well.

Xuan et al. have reported a wearable enzymatic sensor for sweat lactate detection depending on a lactate oxidase (LOx) enzyme [118]. Enzyme immobilization was accomplished by an external polymeric deposit covering the quaternary salt tetra dodecyl ammonium tetrakis (4-chlorophenyl) borate. Moreover, this layer separated the enzyme from direct contact with the sample. During the enzymatic reaction, H_2_O_2_ was produced as a sub-product during the transformation of the lactate to pyruvate in the presence of LOx. Upon applying the activation potential to PB, the PB layer was electrochemically converted to its reduced state and again returned to its oxidation state in the presence of H_2_O_2_. The further conversion happens instantaneously in a significantly negative current at elevated concentrations of H_2_O_2_. The generated H_2_O_2_ was thus responsible for the corresponding electrochemical change of the sensor. The sensor showed a LOD of 1 mM, fast response, reproducibility, and reversibility. The fabricated lactate sensor is apposite for measuring sweat lactate via a microfluidic epidermal patch.

Lin et al. recently developed a hydrogel-based micro patch (THMP), which at the same time acts as a sweat sampling interface along with a medium for electrochemical biosensing of the targeted sweat metabolites [119]. Lactate was chosen as a representative target molecule for characterizing THMP’s performance. The working electrode was comprised of a PVC layer integrated with (i) THMP, as a diffusion-limiting membrane, which improves the dynamic range of the sensor, (ii) a LOx layer, which helped to generate H_2_O_2_ that is proportional to the concentration level of lactate present in THMP and (iii) a PPD layer, which alleviates the interference from the electroactive species in sweat and the MWCNT/PtNP layer, which improves the sensitivity of the sensor due to the high catalytic efficiency and surface area. The reliability of the sweat sample collection method and extraction of sample-to-answer data by combining with an electrochemical sensing system for individual monitoring has been demonstrated herein. To evaluate the potential applicability of the present sensing system, it was combined within a distributed terminal, which requires a fingertip for the real-time collection of sweat sample.

Son et al. have developed a cactus-spine-inspired sweat-sensing patch for healthcare monitoring (Figure 26g) [120]. The shape of the patch network, in combination with the superhydrophobic/superhydrophilic surface constituents, influences the Laplace pressure, which helped in the spontaneous transportation of sweat to the sensing zone, which is beneficial for the continuous monitoring of sweat metabolites. The sweat sensor is comprised of a lactate or glucose oxidase enzyme on the working electrode (WE), an Ag/AgCl electrode as a reference electrode (RE), and a Pt-coated counter electrode (CE). The deposition of the sweat sensor was carried out onto the center of a watch-type substrate. Sensing responses in the presence of sweat lactate and glucose were measured under potentiostatic conditions, which exhibited linear dynamic responses.

Cao et al. have developed a 3D paper-make electrochemical microfluid-based integrated device, namely 3D-PMED, which was modified via wax screen printing [45]. The 3D-PMED is comprised of five layers: a sweat collector, a vertical channel, a transverse channel, an electrode layer, and a sweat evaporator. Furthermore, the electrodes were coupled to the layers of the electrodes in 3D-PMED for the measurement of the perspiration metabolites. Sweat was absorbed in the collector with the help of capillary action. Mechanistically, GOx enhances the rate of the formation of gluconolactone by oxidation of glucose followed by production of H_2_O_2_, which was then electrochemically reduced on a Prussian Blue (PB) surface at a minimal catalytic potential of 0.1 V. The electrochemical outcomes exhibited an excellent linear response for glucose concentrations of 0 to 1.9 mM.

Kim et al. developed a combined cellulose/β-cyclodextrin (β-CD) GOx immobilized enzymatic sensor patch by reverse iontophoresis towards non-invasive screening of glucose levels. In vitro monitoring exhibited a significant diffusion coefficient, which was unaltered even in the presence of interferrants (e.g., sucrose, uric acid, fructose, and acetaminophen) at physiological levels. Cellulose/β-CD/GOx nanofibers (NFs) were highly sensitive to glucose. The diffusion coefficient of glucose in the cellulose/β-CD NFs was investigated by CV. The CV profiles are linear, indicating the diffusion control sensitivity of the sensor. The diffusion coefficient for H_2_O_2_ was roughly found to be 9.0 × 10^−5^ cm^2^ s^−1^, which is also appreciably high [121].

Xuan et al. reported a reduced graphene oxide (rGO) nanocomposite-based working electrode for the sensitive detection of glucose [122]. The Au/rGO/AuPtNP electrode is enclosed with GOx/Nafion. The redox cofactor of GOx enhances the rate of oxidation of glucose to gluconolactone and H_2_O_2_. The generated H_2_O_2_ is amperometrically quantified on the working electrode surface by the redox potential and relates the current to glucose concentration. The H_2_O_2_ will become O_2_, electron and H^+^ ion. A water-repellent band behind the flexible sensor helps in the collection of perspiration and averts delamination of the sensor from the skin. The developed biosensor showed an appreciable amperometric signal to glucose at the concentration range of 0 to 2.4 mM, fast response and good linearity (0.99). The LOD of glucose was evaluated to be 5 µM. The cyclic voltammetry curve indicated that at a potential range of 0.25–0.5 V, an enhancement in the electrochemical oxidation peak current was acquired with an increasing concentration of glucose. Furthermore, a prompt amperometric signal response was obtained at an applied potential of 0.45 V.

Sempionatto et al. have developed a non-invasive skin-worn device for consequent determination of heart rate and blood pressure via ultrasonic transducers and of numerous biomarkers via electrochemical sensors (Figure 26h) [123]. Chemical sensing was apprehended through non-invasive stimulation of sweat at the IP anode, along with ISF extraction at the IP cathode. Lactate, alcohol, and caffeine were screened only in perspiration, while the screening of glucose was carried out only in the ISF. Choronoamperometry was adopted for the electrochemical recognition of the H_2_O_2_ produced from the glucose oxidase, lactate oxidase, and alcohol oxidase catalyzed enzymatic reactions, while DPV was used for the recognition of caffeine.

Moreover, recent research interest in sweat metabolites sensing has been shifted towards designing ultra-sensitive materials combined with excellent electrocatalytic material with conductive materials, such as nanoporous gold. In the domain of sensitive biosensor development, nanoporous gold is advantageous due to its tunable porosity, biocompatibility, chemical stability, ease of preparation, etc. [124,125,126,127] Recently, Lee et al. have reported nanoporous gold combined with fully stretchable capillary microfluidics for developing a wearable biosensor patch for sweat glucose sensing in a non-enzymatic way [128]. The sensor exhibited a good sensitivity of 253.4 µA cm^−2^ mM^−1^ with very good reproducibility. They have exhibited that the variation of surface roughness and surface area of the nanoporous gold electrode would be helpful for enhancing the signal for glucose over other interfering agents.

### 5.4. Wearable Tattoo Sensor

Researchers have developed skin-worn electronic devices that are skin amicable [129]. Such skin-worn lithographic devices are utilized for on-time non-invasive screening of self-healthcare parameters [130]. Epidermal chemical recognition needs direct contact between the wearable electrochemical diagnostic device and the skin. The textile-based screen printed [131] devices are location-specific, and the consequence of washing on such devices is a further challenge that can possibly negotiate with the stability of the sensor during biorecognition of the targeted analytes. To address those apprehensions, researchers are devoted to the development of flexible electrochemical tattoos. Such skin adhesive tattoos may be simply applied to any site on the skin. Tattoo-based sensors are generally single-use devices, and therefore the problem regarding degradation due to washing is not a foremost apprehension [132].

**Figure 27:**
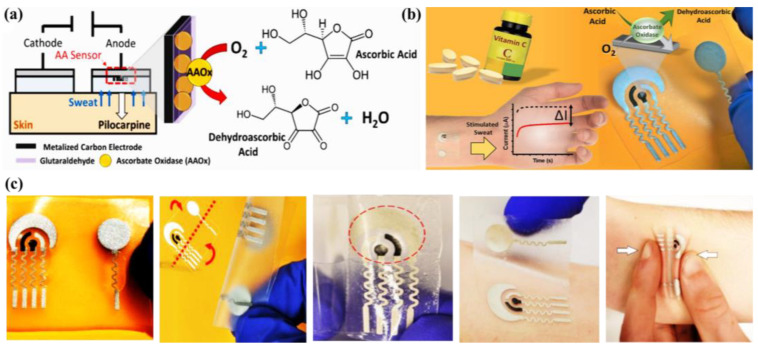
(**a**) Schematic of sweat stimulation using the iontophoretic method and enzymatic reaction for detecting ascorbic acid; (**b**) brief illustration of Vit-C detection by a tattoo-based sensor and corresponding current vs. time plot; and (**c**) skin conformability and mechanical validation of tattoo-based flexible vitamin C biosensor, adopted with permission from Ref. [80].

Sempionatto et al. have fabricated an epidermal sensor for electrochemical recognition of perspiration-derived vitamin C non-invasively by impregnating ascorbate oxidase (AAOx) on stretchable tattoo-based electrodes [80]. The sensor worked on the basis of observing alterations in the level of vitamin C via alteration in the reduction current of the oxygen co-substrate. The tattoo patch for sensing vitamin C was prepared on a polyurethane substrate and integrated into an iontophoretic sweat stimulation system along with amperometric cathodic detection of the oxygen diminution throughout the enzymatic reaction (Figure 27). The enzymatic biosensor showed a discerning response without any interference from electroactive species such as uric acid or acetaminophen. Variations of vitamin C contents in perspiration were checked using different amounts of commercial vitamin C-rich beverages. In addition, the AAOx biosensor was revealed as a single-use strip for in vitro recognition of vitamin C. These observations validate the potentiality of the aforementioned wearable chemical sensors for the non-invasive assessment of nutrition status in the human body.

### 5.5. Wearable Band Sensor

Currently, wearable sweat band-based sensors are widely used for monitoring sweat metabolites as a potential personalized health-monitoring platform [133,134]. This class of wearable sensors is usually worn on the user’s wrist or forearm for on-body sweat analysis in a non-invasive way. Thus, these kinds of sensors are attracting burgeoning research interest due to their growing impact in the contemporary domain of advanced technology integrated with miniaturized devices.

Recently, Jiang and Shen et al. have reported a wearable enzyme-free micro-supercapacitors (MSCs) based sweat glucose sensor, which is comprised of NiCo_2_O_4_ nanoarray/chitosan [135]. The glucose sensing pathway proceeds as follows:

NiCo_2_O_4_ + OH^−^ + 2H_2_O ↔ NiOOH + 2CoOOH + H_2_O + *e*^−^

NiOOH + 2CoOOH + glucose → NiCo_2_O_4_ + glucolactone + 2H^+^ + 2*e*^−^

**Figure 28:**
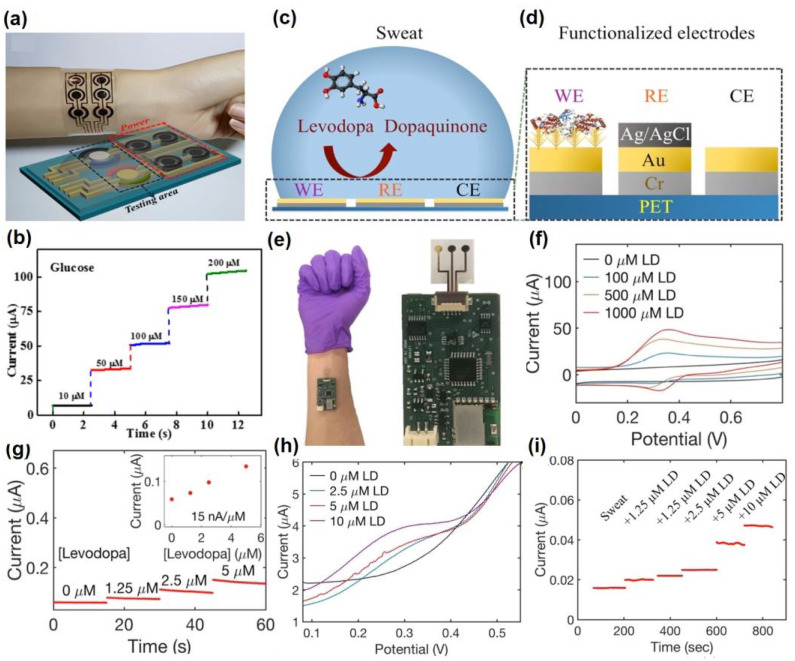
(**a**) Photograph of the wearable wristband integrated with glucose sensor, (**b**) current response of the sensor in presence of increasing concentration of glucose, Adopted with permission from Ref. [135], (**c**) working principle of the electrochemical S-band for monitoring levodopa, (**d**) cross-sectional view of the flexible sensor patch, (**e**) physical image of the S-band worn on user’s wrist, (**f**) cyclic voltammogram data and (**g**) amperometric data of the sensor in presence of increasing concentration of levodopa in buffer medium, (**h**) cyclic voltammogram and (**i**) amperometric data of the sensor in presence of increasing concentration of levodopa (0–10 µM) in sweat. Adopted with permission from Ref. [133].

In the presence of NiCo_2_O_4_, glucose was oxidized to produce glucolactone, and the corresponding redox current was analyzed via anamperometric method. The sensitivity of the electrochemical sensor is much lower towards detection of lactate due to the destruction of the NiCo_2_O_4_ crystal structure, which is not desirable for redox reactions. Herein, the electrochemical signal response from the wearable wristband sensor was transmitted to an individual smartphone via WiFi, which is helpful for self-health monitoring (Figure 28a,b).

Javey et al. have reported a wearable sweat band (S-band) for on-body screening of levodopa metabolism, which is clinically prescribed for Parkinson’s disease (Figure 28c–e) [133]. Levodopa undergoes a xenobiotic metabolism pathway that exhibits a potential correlation of the drug in excreted sweat with that in human blood plasma [136]. Herein, the sweat sensor has been fabricated on a polyethylene terephthalate (PET) substrate, employing a three-electrode configuration, wherein gold nanodendrite functionalized with a tyrosinase enzyme was used as the working electrode, the Ag/AgCl top layer was used as the reference electrode and the Au top layer has been used as the counter electrode. Nafion was drop-casted onto the working electrode to modify the electrode, which thus enhanced the long-term stability and anti-fouling activity of the fabricated sensor. During cyclic voltammetric and amperometric measurements, tyrosinase enzyme facilitated electrochemical oxidation of levodopa to dopaquinone (Figure 28f,g), which generated the Faradic current to trace the concentration of sweat levodopa. The non-invasive levodopa monitoring capability of the electrochemical sensor has been evaluated with sweat specimens, collected from healthy volunteers via iontophoresis and after the consumption of Vicia faba (fava bean) (Figure 28h,i). Nevertheless, the sensitivity of the developed sensor in sweat is less than that in PBS, which may be ascribed to the bio-fouling activity.

**Figure 29:**
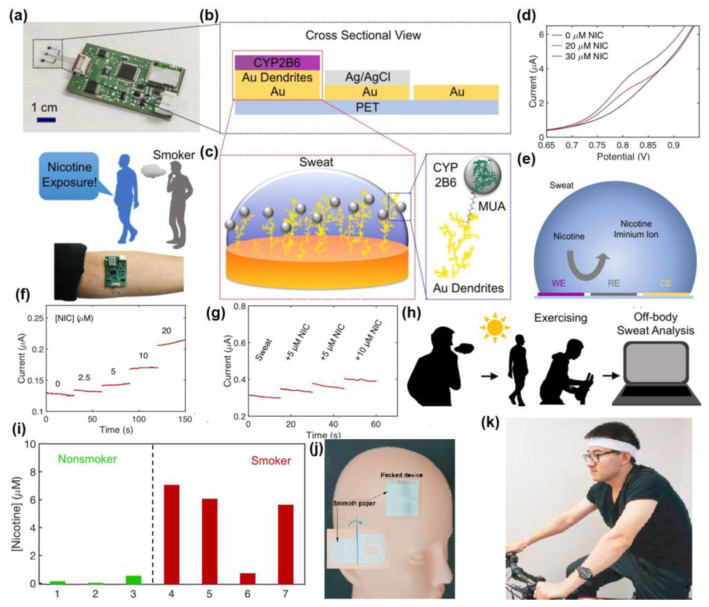
(**a**) Physical image of a wearable sweatband and a schematic illustrating its working principle, (**b**) a cross-sectional view of the flexible sensor patch, (**c**) zoomed-in view of the working electrode, (**d**) cyclic voltammogram and (**e**) working principle of the electrochemical sensor, (**f**) amperometric response of the sensor with increasing concentration of nicotine in PBS, (**g**) amperometric response of the electrochemical sensor in presence of nicotine in sweat, (**h**) schematic illustration of the nicotine monitoring via exercise method, (**i**) concentrations of nicotine, which were measured for smokers and non-smokers, adopted with permission from Ref. [134], (**j**) biosensor attached on the plastic head model before human trial, and (**k**) Sensor patch worn on subject’s body as sweat headband, adopted with permission from Ref. [140].

The same group has again reported a non-invasive wearable sweat band for monitoring sweat nicotine, which is the main ingredient of cigarettes [134]. Currently, direct or indirect tobacco exposure has turned out to be one of the leading epidemics, due to which approximately eight million peoples are suffering worldwide, leading to complex cardiovascular diseases, diabetes, respiratory dysfunctions, etc. [137,138]. This necessitates personalized nicotine monitoring devices for smoker and even non-smoker populations as well. In this regard, Javey et al. have reported a wearable sweat band to be worn on a user’s forearm for nicotine monitoring seamlessly after the inhalation of tobacco (Figure 29a). The electrochemical sensor was comprised of a printed circuit board (PCB) connected flexible electrode array, wherein Ag/AgCl was used as the reference electrode, Au was used as the counter electrode and a gold working electrode was fabricated on a PET film with gold nano-dendrites functionalized with a nicotine oxidizing enzyme, cytochrome P450 2B6, which was covalently linked via 11-mercaptoundecanoic acid (MUA) (Figure 29b,c). From the cyclic voltammogram data, it has been shown that with an increasing concentration of nicotine (0–30 µM), the oxidation current centered at 0.8 V gradually increased in height (Figure 29d) due to the enzymatic electrochemical oxidation of nicotine to a nicotine iminium ion (Figure 29e). The sensitivity of the sensor was also comparable in the sweat solution, as it was in PBS (Figure 29f,g), wherein the sweat specimens were collected from both smoker as well as non-smoker volunteers via cycling, competitive sports and intense walking (Figure 29h,i). The average nicotine concentration in the smokers’ sweat was observed to be 4.8 µM, which may vary from person to person due to variations in metabolic rate, nicotine contents in cigarettes, etc. Thus, the present wearable sweatband will bridge the technological gap between the commercial tobacco detector or tobacco sensing strip due to its on-body measurement capability.

Hamedi et al. have reported a textile-sensing platform for the electrochemical sensing of sweat glucose [139]. The wearable sensor was fabricated via the stitching of threads coated with conductive gold plasma onto fabrics. The gold thread working electrode was functionalized with a thiolate self-assembled monolayer (SAM) integrated with a glucose oxidase (GOx) enzyme and a redox tagged thiol (6-Ferrocenylhexanethiol) for electrochemical regeneration of the enzyme, which would be helpful for continuous glucose monitoring. In the presence of GOx, O_2_ was reduced to H_2_O_2_ and β-D-glucose was oxidized to D-glucono-1,5-lactone. The generated electroactive H_2_O_2_ evolved an oxidation peak at +450 mV vs. Au pseudo reference electrode. Based on the oxidation peak size, the glucose concentration can be measured. The flexible textile sensor can be worn on the user’s hand as a wristband for sweat metabolite detection. The sensitivity of the fabricated sensor is highly analogous to that of conventional ELISA and western blot tests, demonstrating the reliability of the fabricated sensor.

Liu et al. have reported a thread-based wearable e-textile sensor for electrochemical sweat lactate sensing [140]. The sensor was comprised of a conductive threads carbon electrode integrated with zinc-oxide nanowires (ZnO NWs), tetrathiafulvalene (TTF) and lactate oxidase enzymes (LOx). After reaching the positive potential at a certain level, TTF was oxidized to TTF^+^, which acted as a mediator of the lactate sensing process. LOx facilitates the oxidation of lactate to pyruvate with the simultaneous reduction of TTF^+^ to TTF. Based on the concentration ratio of TTF^+^ to TTF in the enzyme membrane, the concentration of lactate can be analyzed. The developed low-cost sensor patch can be worn on the subject’s body as a wearable headband for real-time on-body sweat sensing that can be further wirelessly connected to a smartphone (Figure 29j,k).

Zhang et al. have studied a cloth/paper-based wearable sensor for point-of-care electrochemical monitoring of sweat glucose and lactic acid [141]. Herein, the working electrode was fabricated by screen-printing carbon ink followed by enzymatic functionalization. The sensitivity of the electrode was increased by incorporating the mixture of the multi-walled carbon nanotube (MWCNT) and Prussian blue (PB), which is known as artificial peroxidase. In the presence of GOx, glucose was converted to gluconic acid and H_2_O_2_. MWCNT then helped in electron transfer during the reaction between PB and H_2_O_2_ to generate the corresponding electric signal for glucose selectively. The sensitivity of the fabricated sensor has also been extended with the fabrication of a wearable band for on-body sensing of the sweat metabolites.

Xu and Zhang et al. have reported self-pumping Janus Textile Bands based on an electrospinning hydrophobic polyurethane (PU) nanofiber array for electrochemical on-body sensing of glucose and lactate [142]. A low-cost adhesive tape was used to fix the sensor device onto the human skin to provide a comfortable skin environment, and a bio-compatible polyethylene terephthalate (PET) insulating layer helped to acquire reliable readouts. The Janus textile helped to separate the epidermis from the electrode surface. The electrochemical sensing response was monitored via chronoamperometric measurements, wherein the characteristic peak currents gradually increased with increasing concentrations of glucose and lactate. A hybrid band was developed with the sensor for on-body measurements of the targeted analytes.

### 5.6. Others

**Figure 30:**
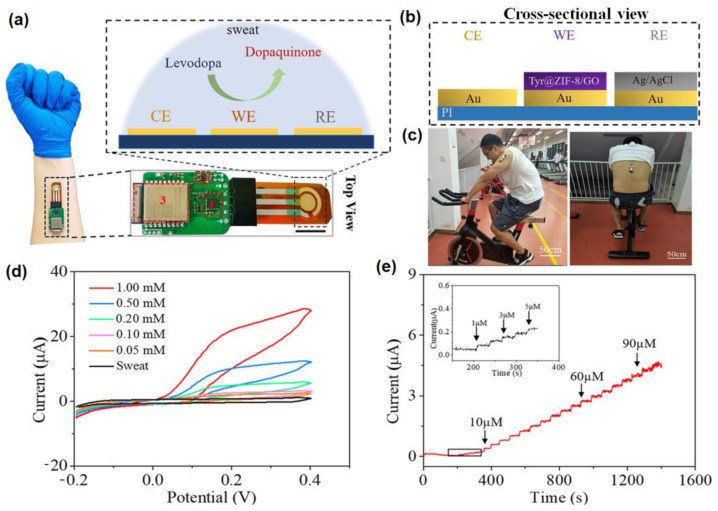
Schematic illustration of (**a**) wearable electrochemical sensor for L-dopa monitoring, (**b**) functionalized electrode fabrication, (**c**) potential application of the L-dopa sensor, (**d**) cyclic voltammogram and (**e**) chronoamperometric data of the sensor in presence of varying concentration of L-dopa. Adopted with permission from Ref. [93].

There are some other types of wearable sensors available for the target-specific detection of sweat metabolites. Xu et al. reported a non-invasive enzymatic sensor comprised of Zeolitic imidazolate framework/graphene oxide composite (ZIF-8/GO) integrated with a tyrosinase enzyme for the electrochemical monitoring of levodopa (L-dopa) in a micromolar range from the sweat specimen [93]. For uninterrupted analysis, a wireless electronic circuit was enabled with the sensor to display the data in a smartphone application via a Bluetooth transceiver. The electrochemical sensor was comprised of a gold working electrode, Ag/AgCl as the reference electrode and Au as the counter electrode. The surface of the working electrode was functionalized with a tyrosinase enzyme, which led to oxidation of L-dopa to dopaquinone, and the corresponding electrochemical signal was monitored. For real-time applicability, the sensitivity of the sensor has also been checked with the sweat specimen from the healthy volunteers after consumption of Vicia faba beans, a natural source of L-dopa (Figure 30).

**Figure 31:**
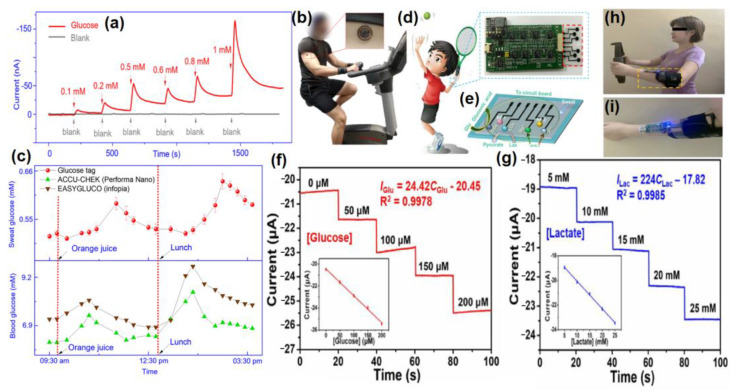
(**a**) Electrochemical response in presence of different concentrations of glucose, (**b**) on-body sweat analysis during physical exercise by the fabricated glucose tag, (**c**) the data of glucose concentration, measured in fabricated glucose tag from sweat and commercially available glucometers from the blood for the time period of 6 h, adopted with permission from Ref. [143], (**d**) photographic image of SWCNT-based flexible sensing array, (**e**) schematic illustration of the multiple sensing of sweat metabolites, amperometric response of the sensor array in the presence of increasing concentration (**f**) glucose and (**g**) lactate, and (**h**,**i**) photographs of the volunteers wearing intelligent wrist band for real-time sweat metabolites analysis, adopted with permission from Ref. [144].

Beker et al. have reported a battery-free wireless tag integrated with a footprint of 1.2 cm^2^ for non-invasive monitoring of glucose [143]. The whole set-up was comprised of microfluidic channels for sweat collection, a glucose-sensitive enzymatic electrochemical sensor, an electronic chip for data processing, an antenna for data transmission to a smartphone and a wireless power supply. The chronoamperometric data exhibited that, with an increasing glucose concentration from 0.1–1 mM, the current response was linearly enhanced. The sensitivity and real-time applicability of the sensor was at first monitored with an artificial sweat sample spiked with a known concentration of glucose, from which glucose was recovered at a quality rate (94.8–96.5%). Then, the in vivo tests were performed with healthy and non-diabetic volunteers after the consumption of orange juice and after having lunch. The sensitivity of the developed glucose tag was comparable with the commercially available fingerstick glucometers, demonstrating the reliability of the present work (Figure 31a–c).

Liu et al. have reported the photosensitive stamp-inspired vacuum filtration-transfer printing method (termed as PS-VFTP) based development of a single-walled carbon nanotube (SWCNT) derived intelligent enzymatic electrochemical sensor array (<$0.33/array) for the simultaneous detection of glucose and lactate (Figure 31d,e) [144]. SWCNT has been chosen due to its high flexibility, stretchability, conductivity, good functionalization capability, etc. [145]. To further improve the conductivity and sensitivity, electrodeposition of Pt nanoparticles was carried out onto SWCNTs surface. Among various strategies of SWCNT-based sensor array fabrication, the vacuum filtration-transfer printing method is advantageous due to its economic benefit and simple operational methodologies. Additionally, by simply changing the filtration solution volume, composition or the template of the filtration membrane, the sensor arrays can be easily customized through this method [146]. The electrode surfaces were functionalized with glucose and lactate oxidase for sensing glucose and lactate, respectively. Prussian blue was used as a catalyst for the effective diminution of the reduction potential of glucose and lactate to ~0 V, thus breaking the necessity of external power supply for operating the sensors. From the amperometric responses, it has been observed that by increasing the concentration of glucose or lactate, the current response was also enhanced (Figure 31f,g). For the real time applicability of the sensor, an “intelligent wristband” was worn on the wrist of a volunteer (Figure 31h,i), and the corresponding signal response could be displayed on a customized smartphone app.

**Figure 32:**
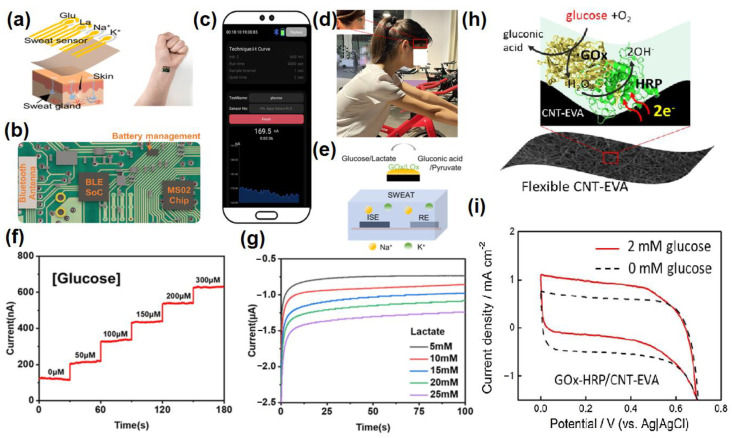
(**a**)Three-dimensional view of the flexible glucose and lactate sensor, (**b**) printed circuit board for glucose and lactate sensing, (**c**) data transmission to smartphone and visualization by the dedicated smartphone APP, (**d**) photograph of human volunteer wearing the sensor during cycling, (**e**) working principle of the sensor array, chronoamperometric response of the sensor array in presence of (**f**) glucose and (**g**) lactate, adopted with permission from Ref. [147], (**h**) schematic illustration of mediator free glucose sensing by GOx/HRP couple and (**i**) the corresponding CV response in presence and absence of glucose, adopted with permission from Ref. [148].

Liu et al. have reported an ultra-small wearable biosensor integrated with a MS02 chip (1.2 mm × 1.1 mm) for on-body detection of sweat metabolites, glucose and lactate [147]. Herein, a rechargeable lithium-ion battery was used for the power supply and the obtained data were transmitted to a mobile phone via Bluetooth. Herein, a thin polyimide substrate was taken for the fabrication of flexible sensor array, wherein a gold layer was taken as the working electrode, which was modified with chitosan/single-walled carbon nanotubes followed by functionalization with lactate oxidase and glucose oxidase for the sensing of lactate and glucose, respectively. In the presence of glucose oxidase, gluconic acid and H_2_O_2_ was produced by the oxidation of glucose, while in presence of lactate oxidase, the lactate was oxidized to pyruvic acid and H_2_O_2_. This resulted in the generation of the corresponding amperometric signals. For on-body analysis of the sweat metabolites by the fabricated chip, the electrochemical data were acquired from human volunteers after cycling (Figure 32a–g). The results were highly comparable with the commercially available potentiostat, demonstrating the reliability of the present work.

Zhang and Zhou et al. have prepared a carbon nanotubes (CNT)-ethylene-vinyl acetate copolymer (EVA) nano-composite-based flexible wearable electrochemical sensor functionalized with glucose oxidase (GOx)-horseradish peroxidase (HRP) for the detection of sweat glucose [148]. Herein, the GOx catalyst helped in glucose oxidation and reduction of O_2_ to H_2_O_2_. HRP then catalyzed the reduction of H_2_O_2_ and took the electrons from the CNT-EVA surface via direct electron transfer (DET) reaction (Figure 32h). Thus, in this process path, the mediator-free glucose determination can be possible. Cyclic voltammogram data exhibited that, with an increasing concentration of glucose, the current density was gradually enhanced (Figure 32i) and reached fast saturation, which may be attributed to fast electron transfer as well as mass transport at the nanostructured enzyme/electrode interface. The sensitivity of the sensor has also been monitored in artificial as well as real sweat specimens.

Huang et al. have reported wearable sensor based on carbon nanotubes (CNTs) and gold nanotubes (Au NTs) forming molecularly imprinted sites for real-time urea electrochemical sensing from human sweat [149]. Herein, the interaction of urea with the molecularly imprinted recognition sites through H-bonding restricts the electron transfer from potassium ferricyanide, leading to selective urea detection. Accordingly, DPV data showed that with an increasing concentration of urea, the current response gradually decreased. The performance of the sensor was also checked with artificial sweat followed by a volunteer’s sweat after aerobic exercise.

Kim et al. have studied a flexible sensor for electrochemical sweat urea sensing based on a flexible NiCu(OOH)/polystyrene (PS) electrode with a carbon nanotube as the conductive element [150]. Herein, PS facilitated the effective electron transfer due to the porous structure. A Ni-Cu alloy acted as the catalyst to facilitate the electro-oxidation of urea in acidic to neutral environments. A 2:1 bimetallic ratio of Ni/Cu achieved the optimum electro-oxidation capability. CV data inferred that with an increasing concentration of current density, the response of Ni_2_Cu_1_(OOH)/PS was gradually enhanced. The flexibility of the electrode made it suitable to be fabricated on cloth for making the wearable sensor. Due to the sensitivity of the sensor for detection of sweat urea, an artificial sweat sample was used for urea detection.

Javey et al. have reported an enzymatic wearable sensor based on LiClO_4_-doped conductive polymer integrated with Au nanodendrites for electrochemical monitoring of sweat ascorbic acid via iontophoresis method with the requirement of minimal volume of sweat (≈1.5 µL) [151]. From the cyclic voltammogram data, it has been observed that the sensor exhibited a sharp oxidation peak at ≈0.2 V. With an increasing AA concentration, the oxidation peak gradually became much more prominent due to the electrocatalytic oxidation of ascorbic acid to the L-ascorbate oxidase. The sensitivity of the sensor was checked from various bio-fluids, such as sweat, urine and blood upon oral intake of vitamin C.

Recently, Ha et al. have reported carbon nanotube (CNT)-Au nanosheets-CoWO_4_-based stretchable electrode for sweat glucose sensing [152]. The stretchable sensor was encapsulated onto a sticky silbione to make it skin-attachable. In the electrochemical window of CV, no oxidation peak was visible in the absence of glucose. However, in the presence of glucose, a new oxidation peak appeared at −0.2 V. Basically, due to electrochemical reaction, CoWO_4_ was transformed to CoOOH followed by CoO_2_, which then led to the oxidation of glucose to gluconolactone that was responsible for the generation of the oxidation peak in the electrochemical window. The profound selectivity of the developed sensor may be ascribed to the electrochemical operation at a low potential, which circumstantially reduces the chance of the oxidation of potentially interfering agents. The sensitivity of the sensor for detecting human sweat was also investigated before and after having a meal and after exercise, which evidently indicates that the results are highly comparable with the commercially available sensor kits.

Xia and Jiang et al. have developed a wearable epidermal enzymatic sensor derived from PANI and single-walled CNT for potentiometric analysis of glucose from human sweat [49]. With the help of a near field communication (NFC) module in the developed device, the generated data could be wirelessly transmitted to any portable reader. Herein, a pH-sensitive electrode was adopted for glucose sensing due to the local pH change in the presence of the glucose oxidase enzyme. The corresponding electrical potential was then measured to quantify the concentration of glucose. Here also, the sensitivity was checked before and after having a meal and after jogging, which would be helpful in the domain of sports and healthcare monitoring.

Li and Ding et al. have reported an electrospun carbon nanofibers (CNFs) based wearable sensor for the electrochemical analysis of sweat uric acid [153]. Herein, CNFs have been chosen because of their porous structure, large surface area, rich active sites for targeted bio-analyte detection, fast electron transfer capability, etc. In presence of uric acid, an obvious redox peak appeared at 0.45 V due to selective oxidation of uric acid. After integrating the developed sensor with commercially available screen-printed electrodes, it was exploited towards the detection of uric acid from sweat by attaching the sensors to different body-parts.

Silva et al. have reported a G. xylinum-based microbial nanocellulose-based wearable skin-like sensor for electrochemical sensing of sweat uric acid [154]. The sensitivity of the bare screen printed carbon electrode is very poor because of the low conductivity due to the limited number of redox-active sites. However, electrochemical pre-treatment increased the number of redox-active oxygenated groups on the carbon surface. Moreover, hydrophilicity was also increased, which cumulatively enhanced the electrical conductivity and electron transfer rate on the carbon surface and lowered the charge transfer resistance (R_ct_).

**Table 3:** Summary of the performances of nanomaterials-integrated wearable electrochemical sensors for the recognition of sweat metabolites.

Operational Platform	Nano Biosensor	Targeted Sweat Metabolites	Sensitivity	Detection Limit	Linear Range	Ref.
Eye Glasses	Bio-enzyme Gel-Membrane	Lactate	0.74 µA mM^−1^	0.04 mM	0–25 mM	[101]
Gloves	Carbonaceous nanomaterials	Uric acid and Drug metabolites (Paracetamol, paroxetine and ethinylestradiol)	-	Uric acid: 1.37 µM Paracetamol:0.247 µM Paroxetine: 0.493 µM Ethinylestradiol: 0.935 µM	Uric acid: 1.0–41.0 × 10^−6^ M Drug metabolites: 1.0–10.0 × 10^−6^ M	[104]
	CNT	Lactate	0.358 µA mM^−1^	2.5 µM	47.6 µM–1.52 mM	[105]
	Enzymatic gold electrode	Ethanol AA	-	-	AA: 0–300 µM	[106]
Patch	Prussian blue-doped poly (3,4-ethylenedioxythiophene nanocomposite (PB-PEDOT NC) electrode	Glucose	-	4 µM	6.25 µM to 0.8 mM	[108]
	Microfluidic sweat patch coupled with AuNPs modified biosensor	Glucose, lactate	Glucose: 24.8 µA mM^−1^ cm^−2^ Lactate: 1.0 µA mM^−1^ cm^−2^	Glucose: 7.34 µM Lactate: 1.24 mM	-	[110]
	Nanohybrid fiber (WSNF) in which Au nanowrinkles partially cover the rGO/PU composite	Glucose	140 µA.mM^−1^ cm^−2^	500 nM	-	[109]
	MIPs-AgNWs biosensor	Lactate	-	0.22 µM	-	[111]
	glucose oxidase coated microneedle-type glucose sensor	Glucose	-	millimolar scale	-	[112]
	Au/PDMS film coated with Ni–Co MOF nanosheet	Glucose	205.1 µA mM^−1^ cm^−2^	4.25 µM	20–790 µM	[113]
	(Ni-Co MOF/Ag/rGO/ PU) fiber-based wearable electrochemical sensor	Glucose	425.9 µA. mM^−1^ cm^−2^	3.28 µM	10 µM−0.66 mM	[114]
	Anti-EtG monoclonal antibodies-based assay	Ethyl glucuronide (EtG), an ethanol metabolite	-	-	10–10,000 ng/L	[115]
	Poly(3,4-ethylenedioxythiophene): poly(4-styrenesulfonate) (PEDOT:PSS) and Pt nanoparticles-based enzymatic sensor	Glucose	325.99 ± 0.8 µA mM^−1^ cm^−2^	10.3 µM	0–2.5 mM	[116]
	HA-AuNP/GOx complex	Glucose	12.37 µA⋅dL/mg⋅cm^2^	0.5 mg/dL	-	[117]
	PB-LOx electrode	Lactate	−641 ± 21 nA mM^−1^	32.6 µM	-	[118]
	Thin hydrogel micropatch with PtNPs deposited MWCNT-modified Au electrode	Lactate	1.88 ± 0.24 µA	0.12 ± 0.02 mM	0−4 mM	[119]
	GOx/Lox-based Cactus-spine-inspired patch	Glucose, Lactate	-	250 µM	-	[120]
	Three-dimensional paper-based microfluidic electrochemical integrated device (3D-PMED)	Glucose	35.7 µA mM^−1^ cm^−2^	5 mM	-	[45]
	Cellulose/-cyclodextrin nanofiber patch	Glucose	5.08 µA mM^−1^	93.5 µM	0–1 mM	[121]
	Reduced graphene oxide (rGO)-based nanostructured composite	Glucose	48 µA mM^−1^ cm^−2^	5 µM	0–2.4 mM	[122]
	Enzyme immobilized Styrene-ethylene-butylene-styrene block copolymer (SEBS)-based stretchable material	Glucose, Alcohol, Lactate, Caffeine	-	-	-	[123]
	Nanoporous gold combined with fully stretchable capillary microfluidics	Glucose	253.4 µA cm^−2^ mM^−1^	-	0.01–1 mM	[128]
Tattoo	Ascorbate oxidase (AAOx) on flexible printable tattoo electrodes	Vitamin C	-	-	0–1000 µM	[80]
Band	NiCo_2_O_4_-based micro-supercapacitors	Glucose, Lactate	Glucose: 0.5 µA/µM Lactate: 0.0075 µA/µM	Glucose: 10 µM	Glucose: 10–200 µM Lactate: 5–25 mM	[135]
	Au nano-dendrites-based enzymatic sensor	Drug metabolite (levodopa)	PBS: 15 nA/µM Sweat: 1.7 nA/µM	Sweat: 1.25 µM	-	[133]
	Au nano-dendrites-based enzymatic sensor	Nicotine	PBS: 4.3 nA/µM Sweat: 4.4 nA/µM	Sweat: 1.6 µM	0–20 µM	[134]
	Au/thiolate self-assembled monolayers-based enzymatic sensor	Glucose	126 ± 14 nA/mM	301 ± 2 nM	0.1–0.6 mM	[139]
	Conductive threads and zinc-oxide nano wires(ZnO NWs) based enzymatic sensor	Lactate	-	3.61 mM	0–25 mM	[140]
	Wearable cloth-based enzymatic sensor integrated with MWCNT and Prussian blue	Glucose, Lactate	Glucose: 105.93 µA mM^−1^cm^−2^ Lactate: 2.28 µA mM^−1^ cm^−2^	Glucose: 4.95 µM Lactate: 0.25 mM	Glucose: 0.05–1 mM	[141]
	Self-pumping Janus textile bands based on electrospinning hydrophobic polyurethane (PU) nanofiber array	Glucose, Lactate	Glucose: 8 nA/µM Lactate: 67 nA/µM	-	Glucose: 0–200 µM Lactate: 0–25 mM	[142]
Others	ZIF-8/GO composite integrated with tyrosinase enzyme	Levodopa (L-dopa)	-	0.45 µM	1–95 µM	[93]
	Glucose oxidase/chitosan/AuNPs-based enzymatic sensor	Glucose	1.27 µA cm^−2^ mM^−1^	24 µM	0.1–1 mM	[143]
	SWCNT-based enzymatic sensor	Glucose, Lactate	Glucose: 345.5 nA mM^−1^ cm^−2^ Lactate: 3169 nA mM^−1^ cm^−2^	-	Glucose: 0–200 µM Lactate: 5–25 mM	[144]
	Chitosan/single walled carbon nanotubes-based enzymatic sensor	Glucose, Lactate	Glucose: 2.054 nA/µM Lactate: 25 nA/mM	-	Glucose: 0–300 µM Lactate: 5–25 mM	[147]
	Carbon nanotubes (CNT) -ethylene-vinyl acetate copolymer (EVA) film coupled with GOx/HRP integrated enzymatic sensor	Glucose	270 ± 10 µA mM^−1^ cm^−2^	Upto 1.0 mM	3 µM	[148]
	carbon nanotubes (CNTs) and gold nanotubes (Au NTs)-based molecularly imprinted sensor	Urea	-	1–100 mM	0.1 mM	[149]
	NiCu(OOH)/polystyrene (PS) electrode with carbon nanotube	Urea	10.72 µAmM^−1^ cm^−2^	2.00–30.00 mM	4.67 µM	[150]
	Nanotextured electrode-based enzymatic sensor	AA	2.0 nA µM^−1^	1000–5000 µM	≈4 µM	[151]
	CNT-Au nanosheets-CoWO_4_ based stretchable sensor	Glucose	10.89 µA mM^−1^ cm^−2^	1.3 µM	0–0.3 mM	[152]
	PANI and single-walled CNT-based enzymatic sensor	Glucose	-	0.1 mM	0.1–50 mM	[49]
	Electrospun carbon nanofibers (CNFs)-based wearable sensor	Uric acid	-	-	10–400 µM	[153]
	*G. xylinum* derived microbial nanocellulose-based wearable skin-like sensor	Uric acid	0.18 A.M^−1^	1.8 µM	0–70 µM	[154]

## 6. Conclusions

Human sweat is comprised of a number of important metabolites, which may act as significant biomarkers for various human diseases. The concentration of sweat metabolites has a direct correlation with those in blood. Thus, the detection of sweat metabolites may non-invasively derive the biochemical information of human health status, therefore reducing the stress of invasive blood tests. Sweat metabolite sensing with the aid of nanomaterials-based wearable technology has garnered remarkable research interest in the regime of non-invasive, personalized health-status monitoring. In this review, we have schematically introduced the basics of the electrochemical sensing mechanism (e.g., amperometry, potentiometry, CV, DPV, SWV and EIS) responsible for targeted sweat metabolites sensing by nanomaterials, which will be beneficial for the fundamental understanding of the electrochemical sensing. Then, recent research endeavors of the last five years (2018–2022), containing a plethora of nanosensors aimed toward the electrochemical sensing of various sweat metabolites, (e.g., lactate, glucose, urea/uric acid, ascorbic acid, ethanol) have been briefly reviewed, scrutinizing the corresponding sensing mechanism in each individual section. Along with the monitoring of the sweat metabolites, the focus has also been levied towards sensing drug metabolites, which will provide an added impetus towards investigating drug abuse. Moreover, the sensing performance of the developed nanosensors has been discussed in light of their detection thresholds, linear dynamic ranges, mode of sensing, etc., which might be regarded as figures of merit of the reviewed sensor materials. This discussion will offer a fundamental rationale for the judicious designing of a sweat metabolite sensor with promising sensitivity. Most of the enzymatic biosensors for sweat metabolites sensing suffer from poor stability, easy inactivation, unsuccessful immbolization, etc. In view of the above, herein the integration of the nanomaterials with the electrochemical sensors has been focused to improve the sensor stability and sensing performance. Due to having electrocatalytic behavior along with excellent conductive properties, nanomaterials-based electrochemical sensing has now become a hot topic in sweat metabolite sensing. The surface of the working electrode can be simply chemically modified by nanomaterials to initiate the electrocatalytic reactions of the sweat metabolites at a particular bias voltage to produce the corresponding current signal for quantification of the guest analytes. One step ahead, the synchronization of the electrochemical sensors with flexible electronics makes a suitable platform for developing wearable electronics in the form of various wearable platforms, such as eyeglasses, tattoos, wristbands, headbands, gloves, patches, etc., for on-body measurements of the sweat metabolites quantitatively, which are beneficial for rapid on-time monitoring of the health status. However, the developed wearable sensors are still in their infant stage. Therefore, in spite of significant advancements in the domain of nanosensing for the recognition of sweat metabolites, there is still much room for improving the performance of the designed sensors. In the present review, the loopholes of the current research endeavor and the corresponding futuristic aspects have also been outlined, which would be helpful for the strategic development of more sensitive sensors for the next-generation paradigm in the field of personalized health monitoring.

## 7. Outlook and Future Prospects

In the present review, the advancement of sweat metabolites sensing by electrochemical nanosensors has been elaborately discussed with their corresponding sensing phenomenon. Both enzymatic as well as non-enzymatic sensors have been focused on for expeditious sensing of the targeted sweat metabolites. Despite the considerable advancements in wearable chemical sweat sensors, numerous challenges persist in the characteristics associated with the operation, stability, sensitivity, selectivity, biofouling, and power source that must be focused on for assessing the wearable sweat sensors as a promising platform for personalized health monitoring. In most cases, the fabricated devices are in the proof-of-concept prototype stage. The main challenges, which deserve future research attention, are depicted below:

(i) Sensitivity and specificity: Due to the presence of sweat metabolites at the trace level, ultrasensitive sensors with preconcentration techniques are obligatory. There is a plethora of literature on non-enzymatic sweat sensing. However, in most cases, they suffer from selectivity issues. Interfering agents and non-specific binding may cause signal alteration, producing a false-negative or -positive response. This indicates that sensor specificity is one of the most crucial parameters, which requires further improvement by improvising judiciously designed nanomaterials with enhanced surface areas and improved electrocatalytic activity.
(ii) Simultaneous analysis of multiple sweat analytes: The currently developed sensors are mostly aligned towards monitoring limited numbers of sweat metabolites. Therefore, wearable sensors may be fabricated in such a way that they should be capable enough for simultaneous monitoring of multiple analytes in sweat, as this strategy offers cost-effective, and less time and sample-consuming responses. In this regard, a microfluidics strategy may be integrated for fast in situ detection of the targeted biomarkers present in sweat specimens. This will offer a comprehensive understanding of the wearer’s health status.
(iii) Circumvention of sensor calibration: Most of the potentiometry-based wearable chemical sweat sensors necessitate calibration procedures, conditioning solutions storage, and other cumbersome procedures. Moreover, the on-field application of these devices entails a stable response over a wide range of temperatures, sunlight, sweat compositions, and other issues that might alter the sensing response. These inconsistencies are important due to the extremely low concentrations of the targeted metabolites present in sweat. Herein, interfering species could further lessen the sensing performance of the fabricated sensor. In this context, for large-scale production, wearable devices should be self-calibrated, as the sensitivity is largely affected by various environmental factors, such as temperature, humidity, sweat collection spots, etc. Therefore, calibration-free or factory-calibrated sensors are gaining much more commercial acceptance.
(iv) Circumvention of multistep sensing: Traditional mechanized biosensors adopt multistep sensing mechanisms for the detection of the analytes at ultralow levels. Moreover, sample pre-treatments are also necessary to improve the selectivity of the sensor. However, these kinds of techniques are not compatible with wearable devices, as in these platforms, sweat specimens must be collected directly from the skin to produce an in situ signal. Hence, newly developed wearable electrochemical sensors for highly sensitive and selective sweat metabolite sensing, which are stable over various environmental conditions, must avoid multistep sensing.
(v) Direct contact with sweat: Difficulties can also arise from the processes of fabrication, and materials, wherein various components must be encapsulated. For instance, chemical sensors should remain in direct contact with sweat, although supportive electronics must be entirely wrapped and should be separated from any biofluid or moisture contact. The contamination of sweat from the exterior of the skin or from the skin adjacent can hardly be eliminated after sweat secretion and in skin contact, which has a huge impact on data correctness. Furthermore, plain washing of the skin surface cannot alleviate bacterial contamination on the skin and may create substantial faults. One possible solution may be the isolation of sweat from the skin surface by coating it with a barricading layer of an oil-based substance on the skin surface.
(vi) Sampling with improved rates of sweat: At present, hard work or iontophoresis is the major approach to stimulate the generation of sweat. However, sweat secretion rates will not exceed 20 nL/min/gL, even after exhaustive exercise. Locally stimulated sweating via an iontophoresis method is more suitable for infants, aged persons, etc., for obtaining enough sweat specimens in inactive conditions in comparison to the condition of exercise. However, controlling the current density of the device should be properly carried out, as repetitive application of an iontophoretic current at the same spot may be harmful to skin. Therefore, the development of ultrasensitive sensors with better sensitivity with a low volume of sweat will be highly promising.
(vii) Scale-up manufacturing of the sensors: A technological emphasis is also required for scale-up manufacturing of the sensors. Commercial-scale manufacturing not only requires a highly feasible fabrication strategy, but should also be relatively cost-effective.
(viii) Reproducibility and durability: In most wearable devices, adhesives are used, but these last only for a few days due to several factors, such as skin oils, irritation, baths, etc. Additionally, in most cases, there occurs consumption or degradation of the chemical probes used in sensors over time. Hence, long-term reusability and durability of the sensors in the complex biological matrices are of immediate need that are reliant on the fabrication of advanced materials and subsequent manufacturing.
(ix) Difficulty in detection due to the complexity of the human body: Keeping in mind the complications of the human body, skin-interfaced flexible systems may identify different biomolecular levels and vital signs, representing a fruitful solution for precisely forecasting and recognizing more explicit health circumstances. Research in these commands will have a noteworthy impact on personalized healthcare.
(x) Better accuracy in sensing response: The judicious designing of more new electrode materials is of tremendous importance to obtain more accurate signal responses in the presence of harsh environmental factors (e.g., high temperature, high humidity, variation in pH, etc.) and to avoid motion falsification during movement.
(xi) Biocompatibility: One crucial parameter of wearable sweat sensing is the development of biocompatible sensors. As the wearable sensors remain tightly in contact with the skin for a long period of time, the sensor material should be biocompatible in nature along with its intrinsic sensing characteristics to avoid skin allergies or other skin-related issues after long-term uses of the wearable sensors. In most of the cases, researchers are focused toward the development of sensors with improved flexibility, stretchability or conductivity, sacrificing the biocompatibility of sensor materials. Hence, considerable research attention should be paid to the development of biocompatible sensor material.
(xii) Mechanical and operational stability for wearable sensors: Mechanical and operational stability of the developed sensors is of paramount importance to obtain a reliable response signal for their applicability during longer operational time periods. Therefore, special attention should be levied on the issues of surface biofouling, enzymatic stability, etc., for longer operational stability.
(xiii) Enhancement of user comfort: Further research attention may be levied to increase the user comfort of wearable devices. To solve the issue, development of soft flexible electronics may be taken into consideration to suit the skin contours and to enhance the wearers’ comfort with the device mainly with the interface of the skin and wearable electronics.
(xiv) Requirement of continuous power supply: Equal challenges also exist in technological interventions for continuously powering wearable chemical sensors. Most wearable devices exploit commercial batteries despite their weight, bulk, and mechanical properties. However, flexible batteries have some potential in this context, but at a significant added price. Research examples of wearable flexible batteries are of interest, but most of them have mediocre performance. In this context, battery-free strategies, based on wireless power transfer using near-field communication (NFC) technologies, are much more advantageous, but need adjacent propinquity (up to ∼1 m) to a transmission antenna for the interminable acquisition of data. Eventually, an efficient combination of various subsystems is required in wearable sweat sensors, which can overcome this technological hurdle.
(xv) Validation: After meeting the above unmet criteria and obtaining confirmatory data from centralized lab tests, the sensing performance of the developed sensors in terms of accuracy and precision should be clinically validated against gold standard LC-MS methods for widespread commercial applications, as slight positive or negative signal predictions will lead to serious life-threatening issues.

With the development of selective sweat metabolite sensors along with the integration with suitable wearable electronics, it is anticipated that the developed wearable sensors are gradually maturing for commercialization in the realm of fitness, sports, personalized health status monitoring and related domains. Although numerous multidisciplinary efforts are entailed for addressing the difficulties before widespread commercialization of the developed proof-of-concept prototype devices, we do believe that the developed wearable sensor materials will offer great impacts in the regime of interdisciplinary fundamental sciences, congregating sensors, clinical diagnosis, wearable electronics, data transmission, and machine learning approaches for processing the medical information to ultimately acquire an advanced personalized healthcare device with desirable performance.

## Figures and Tables

**Table 1:** The main metabolites present in sweat, their concentration in sweat and the related diseases.

Sweat Metabolites	Relative Content	Related Health Condition	Ref.
Glucose	10–200 µM	Diabetes	[25]
Urea/uric acid	2–10 mM	Renal dysfunction, Gout	[26]
Lactic acid	5–20 mM	Stress Ischemia, Cystic Fibrosis	[27]
Ascorbic acid	10–50 µM	Kidney Disease, Thrombosis, Cancer	[28]
Ethanol	2.5–22.5 mM	Alcoholism, Diabetes, Drunk driving	[29]

## Data Availability

Not applicable.
